# Effect of rhythmic auditory cueing on parkinsonian gait: A systematic review and meta-analysis

**DOI:** 10.1038/s41598-017-16232-5

**Published:** 2018-01-11

**Authors:** Shashank Ghai, Ishan Ghai, Gerd Schmitz, Alfred O. Effenberg

**Affiliations:** 1 Institute of Sports Science, Leibniz University Hannover, Hannover, Germany; 2 School of Life Sciences, Jacobs University Bremen, Bremen, Germany

## Abstract

The use of rhythmic auditory cueing to enhance gait performance in parkinsonian patients’ is an emerging area of interest. Different theories and underlying neurophysiological mechanisms have been suggested for ascertaining the enhancement in motor performance. However, a consensus as to its effects based on characteristics of effective stimuli, and training dosage is still not reached. A systematic review and meta-analysis was carried out to analyze the effects of different auditory feedbacks on gait and postural performance in patients affected by Parkinson’s disease. Systematic identification of published literature was performed adhering to PRISMA guidelines, from inception until May 2017, on online databases; Web of science, PEDro, EBSCO, MEDLINE, Cochrane, EMBASE and PROQUEST. Of 4204 records, 50 studies, involving 1892 participants met our inclusion criteria. The analysis revealed an overall positive effect on gait velocity, stride length, and a negative effect on cadence with application of auditory cueing. Neurophysiological mechanisms, training dosage, effects of higher information processing constraints, and use of cueing as an adjunct with medications are thoroughly discussed. This present review bridges the gaps in literature by suggesting application of rhythmic auditory cueing in conventional rehabilitation approaches to enhance motor performance and quality of life in the parkinsonian community.

## Introduction

Susceptibility to fall grows rapidly amongst elderly and patients with neurological deficits^1–3^. The impairments in neuromuscular functioning promotes instability^4,5^, weakness^6^, reduces physical activity^7^, further leading to musculoskeletal deformities and a higher predisposition to fall^8^. Injuries related to such instabilities inflict heavy costs at both individual and economic levels^9^, increase dependency, social isolation and affects the quality of life^10,11^. Neurological disorders such as parkinsonism presents itself with impairments in motor functions exhibiting characteristics such as, akinesia, chorea, hypokinesia, bradykinesia, motor blocks, rigidity, and problems with generation of cyclic movements, further leading to “freezing” instances^12,13^. Jankovic and Tolosa^14^ suggests the degeneration of dopaminergic cells in substantia nigra in basal ganglia, which might result in its impaired excitatory output and affect its functioning (autonomic control of movement planning, scaling and initiation)^15^. Likewise, ageing together with parkinsonism results in rigorous denervation and re-innervation due to progressive reduction in functional motor units in spinal cord and myelinated ventral root fibers^16^. Together, these neurological dysfunctions impair the ability to execute and maintain autonomic motor tasks such as, posture and gait^17^.

Research also suggests a “fear” related stability modification in gait, postural performance for such fall prone population groups^18^, possibly leading to a range of spatiotemporal and kinematic modifications^19^. For instance, reduction in gait velocity, stride length, increase in double limb support^8,18,20^, and stride-to-stride fluctuation^21^ have been extensively reported. This compensatory effort to develop gait patterns that are resistant to external perturbations leads to poor static and dynamic stability^8,22^. Practically, these changes might impair an individual’s ability to pass safely through high stress situations constrained by space or time, such as escalators, traffic signals^4,23^, leading to an increased predisposition to fall. Studies have suggested that this modification in gait patterns is due to an alleviation in “internal” conscious attention towards autonomic control, which adversely impacts proprioception and autonomic functioning possibly because of movement specific re-investment^1,24,25,26^. The theory suggests that directing attention internally to control autonomic movements such as gait, can have an adverse impact on its performance^1^. The theory further adds that aging^23^, neurological ailment and injuries^1,3^ are common conditions that promote movement specific reinvestment. Such fall-prone population groups have a differential cortical activation pattern, which could possibly be linked with changes in task prioritization and conscious attention while carrying out cognitive or motor tasks^27^. Moreover, electromyographic analysis has revealed enhanced variability in motor unit recruitments that adversely impacts the execution of automated and voluntary motor tasks^28^. Likewise, limitations in execution of functional activities of daily living tasks have also been extensively reported^29–32^.

Common treatment strategies to curb motor dysfunctions in parkinsonism include training with virtual-reality^33^, biofeedback^34^, physical/occupational therapy^35^, physical exercise^36^, dance^37^, treadmill^38^, external sensory feedback^39^, and dual-task training^4^. Likewise, pharmacological intervention with psychoactive drug such as levodopa, dopamine agonists, monoamine oxidase type B inhibitors^40^, have been reported to be effective short-term for managing motor symptoms, such as bradykinesia, tremors^41^. However their effectiveness in managing gait and postural dysfunctions in long-term is largely debated^42,43^.

Spaulding *et al*.^43^ argued that the lack of adequate sensory information in patients with parkinsonism plays a destructive role in autonomic motor functioning. Therefore, motor performance in parkinsonian patients might benefit from additional sensory information. Several studies have analyzed the effects of augmented external auditory, visual and tactile feedback on performance^44–47^. Nevertheless, studies have suggested the predominant role of auditory information as compared to its counterparts^39,43^. Predominantly auditory cortex has been reported to perceive stimuli with shorter reaction times (20–50 ms) as compared to its visual or tactile counterparts^45,48–50^. Further, the auditory cortex possesses rich connectivity to motor centers from spinal cord extending towards brainstem, cortical and subcortical structures^51–53^. Thereby, allowing strong cross-sensory impacts of auditory signal characteristics, such as frequency^54^, timbre^55^, on motor execution. Consequently, several types of external auditory feedback techniques have been analyzed in the literature, such as rhythmic auditory cueing^53^, patterned sensory enhancement^46,56^, and real-time auditory feedback^57^. However, rhythmic auditory cueing is most widely studied, with respect to motor performance post parkinsonism^45^, stroke^58^, cerebral palsy^59,60^, and more^20,61–63^. Rhythmic auditory cueing is defined as a medium of repetitive isosynchronous beats applied with an aim to synchronize motor execution with a rhythm^53,58^. The underlying mechanisms for attaining benefits in the motor domain are suggested to be multifactorial^45,64^. The auditory cueing has been suggested to modulate neuromagnetic β oscillations^65^, enhance biological motion perception^57,66^, promote motor imagery^67,68^, reducing shape variability in musculoskeletal activation patterns^69^, mediate cortical reorganization, neural-plasticity^70^, supressing movement specific re-investment^71^, and more^72,73^.

We identified high quality systematic reviews analysing the effects of external auditory cueing on Parkinsonism^42,43,74^. However, the meta-analysis due to extremely strict inclusion criteria allowed the inclusion of only randomized controlled trials for statistical analysis, and not for a joint qualitative analysis^42,43^. Moreover, findings from the meta-analysis of Spaulding *et al*.^43^ were interpreted without the presence of any heterogeneity test in between the studies. Similarly, limitations concerning statistical analysis were observed for Rocha *et al*.^42^. Lim *et al*.^74^ and Nombela *et al*.^45^ performed excellent quality narrative reviews, but the lack of statistical analysis doesn’t allow to draw firm conclusions. Moreover, none of the review studies analysed the effects of different types of tempo, different signal characteristics, training dosage, and dual-task performance with rhythmic auditory cueing. Therefore, we attempted to develop a state of knowledge for the benefit of parkinsonian patients and medical practitioners, where both qualitative and quantitative data from good quality studies can be interpreted. Moreover, to the best of our knowledge, up to now, no review has elucidated the effects of dual tasks, fast/slow paced stimuli, and the precise training dosage of rhythmic auditory cueing on spatiotemporal gait parameters in Parkinson’s disease. This present review for the first time, conducted a systematic review in combination with a meta-analysis to determine the effects of rhythmic auditory cueing among parkinsonian patients.

## Methods

This review was conducted according to the guidelines outlined in Preferred Reporting Items for Systematic Reviews and Meta-analysis: The PRISMA statement^75^.

### Data sources and search strategy

Academic databases Web of science, PEDro, EBSCO, MEDLINE, Cochrane, EMBASE and PROQUEST were searched from inception until July 2017. A sample search strategy has been provided in (Table 1).

**Table 1:** Sample search strategy on EMBASE database.

DATABSE	EMBASE
DATE	10/07/2017
STRATEGY	#1 AND #2 AND #3 AND #4 AND #5 AND #6 AND #7
**#1**	(‘rhythmic auditory feedback’ OR ‘rhythmic auditory cueing’ OR ‘rhythmic acoustic feedback’ OR ‘rhythmic auditory entrainment’ OR ‘metronome feedback’ OR ‘metronome’ OR ‘rhythmic metronome feedback’ OR ‘acoustic stimulus’ OR ‘acoustic feedback’ OR ‘acoustic cueing’ OR ‘external stimuli’ OR ‘external feedback’ OR ‘external cueing’ OR ‘music therapy’ OR ‘Neurological music therapy’ OR ‘tempo’ OR ‘beat’ OR ‘rhythm’ OR ‘RAC’ OR ‘NMT’)/de OR (rhythmic auditory feedback OR rhythmic auditory cueing OR rhythmic acoustic feedback OR rhythmic auditory entrainment OR metronome feedback OR metronome OR rhythmic metronome feedback OR acoustic stimulus OR acoustic feedback OR acoustic cueing OR external stimuli OR external feedback OR external cueing OR music therapy OR Neurological music therapy OR tempo OR beat OR rhythm OR RAC OR NMT)ti,ab
**#2**	(‘Parkinson’s disease’ OR ‘Parkinsonism’ OR ‘Parkinson disease’ OR ‘Parkinson’ OR ‘Parkinson’s’ OR ‘PD’)/de OR (Parkinson’s disease OR Parkinsonism OR Parkinson disease OR Parkinson OR Parkinson’s OR PD); ti,ab
**#3**	(‘cognitive task’ OR ‘concurrent task’ OR ‘dual task’ OR ‘dual task’ OR ‘dual task paradigm’ OR ‘dual task paradigm’ OR ‘cognitive task training’ OR ‘dual task training’ OR ‘dual task training’)/de OR (cognitive task OR concurrent task OR dual task OR dual task OR dual task paradigm OR dual task paradigm OR cognitive task training OR dual task training OR dual task training’):ti,ab
**#4**	(‘rehabilitation’ OR ‘treatment’ OR ‘rehab’ OR ‘management’ OR ‘therapy’ OR ‘physiotherapy’ OR ‘physical therapy’ OR ‘prevention’ OR ‘risk prevention’)/de OR (rehabilitation OR treatment OR rehab OR management OR therapy OR physiotherapy OR physical therapy OR prevention OR risk prevention);ti,ab
**#5**	(‘walking’ OR ‘gait’ OR ‘locomotion’ OR ‘range of motion’ OR ‘ROM’ OR ‘ambulation’ OR ‘mobility’ OR ‘treadmill gait’ OR ‘balance’ OR ‘stability’ OR ‘stride’ OR ‘gait training’ OR ‘gait rehabilitation’)/de OR (walking OR gait OR locomotion OR range of motion OR ROM OR ambulation OR mobility OR treadmill gait OR balance OR stability OR stride OR gait training OR gait rehabilitation);ti,ab
**#6**	(‘age groups’ OR ‘adolescent’ OR ‘young’ OR ‘elderly’ OR ‘old’ AND (‘gender’ OR ‘male’ OR ‘female’) AND (‘athlete’ OR ‘elite athlete’ OR ‘recreational athlete’ OR ‘novice athlete’ OR ‘trained athlete’ OR ‘sedentary’))/de OR (age groups OR adolescent OR young OR elderly OR old AND (gender OR male OR female) AND (athlete OR elite athlete OR recreational athlete OR novice athlete OR trained athlete OR sedentary));ti;ab
**#7**	clinical trial/exp OR (‘intervention study’ OR ‘cohort analysis’ OR ‘longitudinal study’ OR ‘cluster analysis’ OR ‘crossover trial’ OR ‘cluster analysis’ OR ‘randomized trial’ OR ‘major clinical study’)/de OR (longitudinal OR cohort OR crossover trial OR cluster analysis OR randomized trial OR clinical trial OR controlled trial);ti,ab

### Data extraction

Upon selection for review, the following data were extracted from each article; author, date of publication, sample size, sample description (gender, age, health status), disease duration, intervention, characteristics of auditory cueing, dual-task, outcome measures, results, and conclusions. The data were then summarized and tabulated (Supplementary Table 1).

The inclusion criteria for the studies was (i) Randomized controlled trials, cluster randomized controlled trials or controlled clinical trials; (ii) Studies reporting reliable and valid spatiotemporal gait parameters (iii) Studies including static/dynamic aspects of gait/postural stability (iv) Studies scoring ≥4 in PEDro methodological quality scale; (v) Experiments conducted on human participants; (vi) Published in a peer-reviewed academic journal; (vii) Articles published in English, German and Korean languages.

### Quality and risk of bias assessment

The quality of the studies was assessed using the PEDro methodological quality scale^76^. The scale consists of 11 items addressing external validity, internal validity, and interpretability and can detect potential bias with fair to good reliability^80^, and validity^76^. A blinded rating of the methodological quality of the studies was carried out by the primary reviewer (SG). Ambiguous issues were discussed with second and third reviewers (IG, GS, AOE) and consensus was reached. Included studies were rated according to scoring of 9–10, 6–8 and 4–5, and were interpreted as “excellent”, “good” and “fair” quality studies^78^, respectively. Inadequate randomization, non-blinding of assessors, no intention to treat analysis and no measurement of compliance were considered as major threats for biasing^79^.

### Data Analysis

This systematic review also included a meta-analysis approach to develop a better understanding of the incorporated interventions^80^. The presence and lack of heterogeneity asserted the use of either random or fixed effect meta-analysis^81^. A narrative synthesis of the findings structured around the intervention, population characteristics; methodological quality (Supplementary Table 1) and the type of outcome are provided. Likewise, summaries of intervention effects for each study were provided in a tabular format (Supplementary Table 1). A meta-analysis was conducted between pooled homogenous studies using CMA (Comprehensive meta-analysis V 2.0, USA). Heterogeneity between the studies was assessed using I^2^ statistics. The data in this review were systematically distributed and for each available variable pooled, dichotomous data were analyzed and forest plots with 95% confidence intervals are reported. The effect sizes were adjusted and reported as Hedge’s g^82^. Thresholds for interpretation of effect sizes were as follows; a standard mean effect size of 0 means no change, negative effect size means a negative change, mean effect size of 0.2 considered a *small* effect, 0.5 a *medium* effect and 0.8 a *large* effect^83^. Interpretation of heterogeneity via I^2^ statistics was as; 0%, 25%, 75% as negligible, moderate and substantial heterogeneity, respectively. Meta-analysis reports indicating heterogeneity among studies were evaluated to determine the reason of heterogeneity, and the included studies were then pooled separately and analyzed again. The alpha level of 0.05 was adopted.

## Results

### Characteristics of included studies

**Figure 1:**
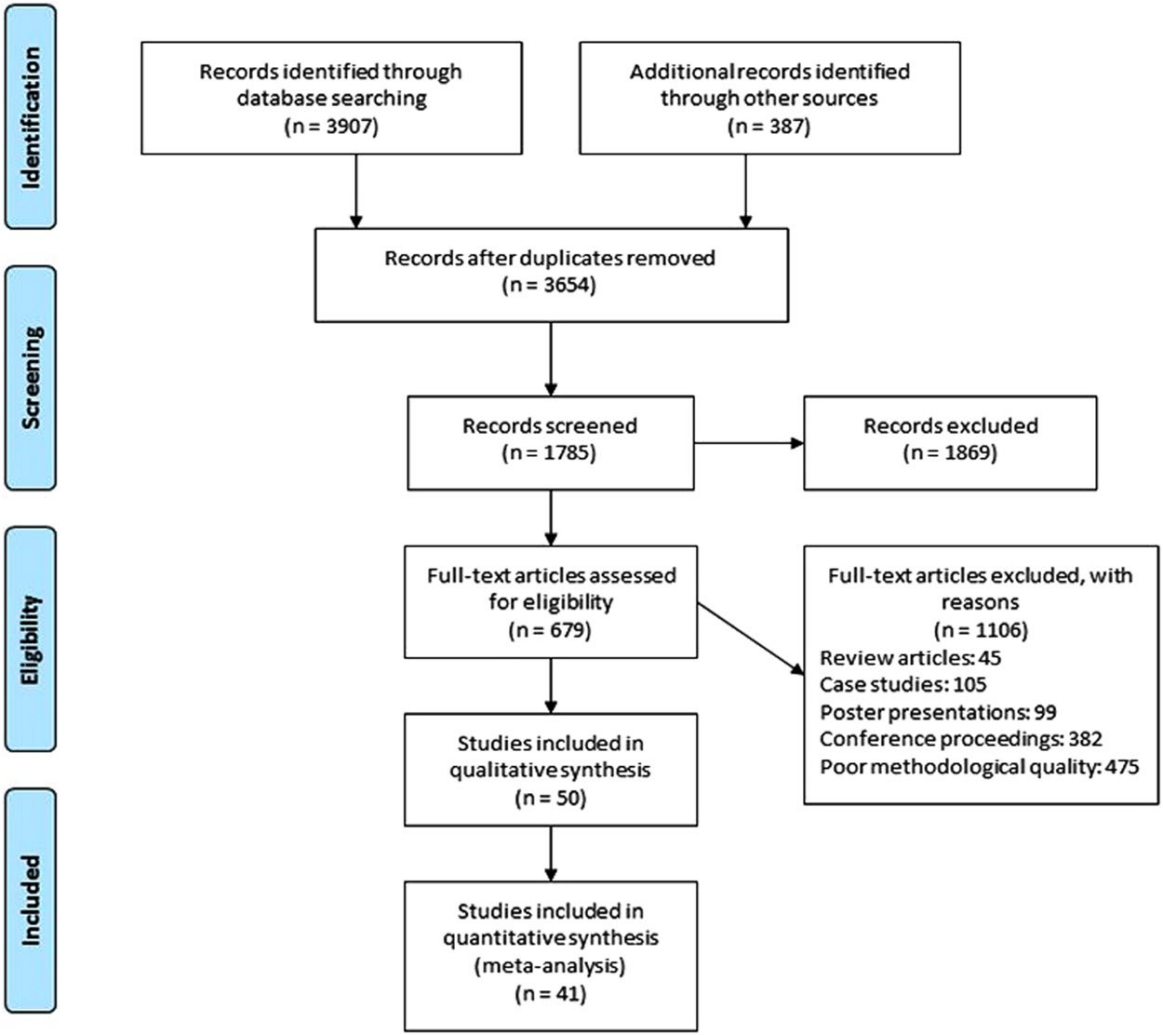
PRISMA flow chart for the inclusion of studies.

Our initial search yielded a total of 4794 studies, which on implementing our inclusion/exclusion criteria, were reduced to fifty (Fig. 1). Data from the included studies have been summarized in (Supplementary Table 1). Of the fifty included studies, seven were randomized controlled trials, and forty-four were controlled clinical trials.

#### Participants

A total of 1892 participants were analyzed in the incorporated studies. In the included studies, forty-eight studies incorporated mix gender patients. Two studies incorporated only male participants^84,85^. Two studies didn’t specify the gender of the included participants^86,87^. The included studies provided data on 1892 participants (n = 745 females/1089 males). Descriptive statistics relating to the age (mean ± standard deviation) of the participants were tabulated across the studies. In addition, the age of participants was mentioned in range by six studies^88–93^, and only a mean value was provided by two studies^31,94^. Disease duration of parkinsonian patients have been mentioned (see Supplementary Table 1).

#### Risk of bias

**Figure 2:**
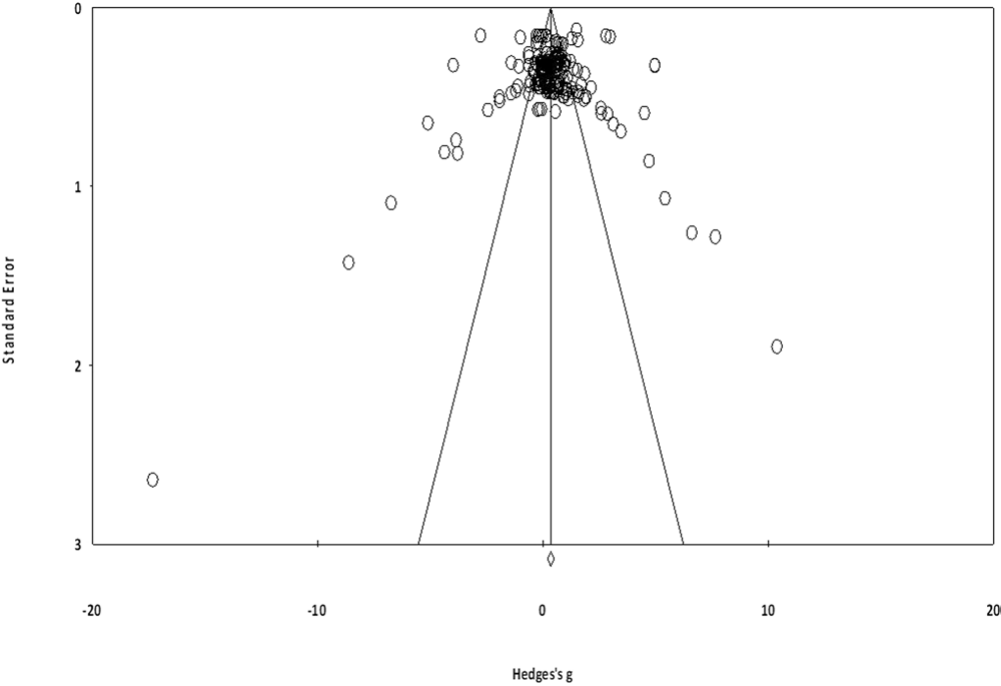
Funnel plot for Hedge’s g and standardized effect for each value in the meta-analysis. Each of the effect is represented in the plot as a circle. Funnel boundaries represent area where 95% of the effects are expected to lie if there were no publication biases. The vertical line represents the mean standardized effect of zero. Absence of publication bias is represented by symmetrically distributed effects around the line.

**Figure 3:**
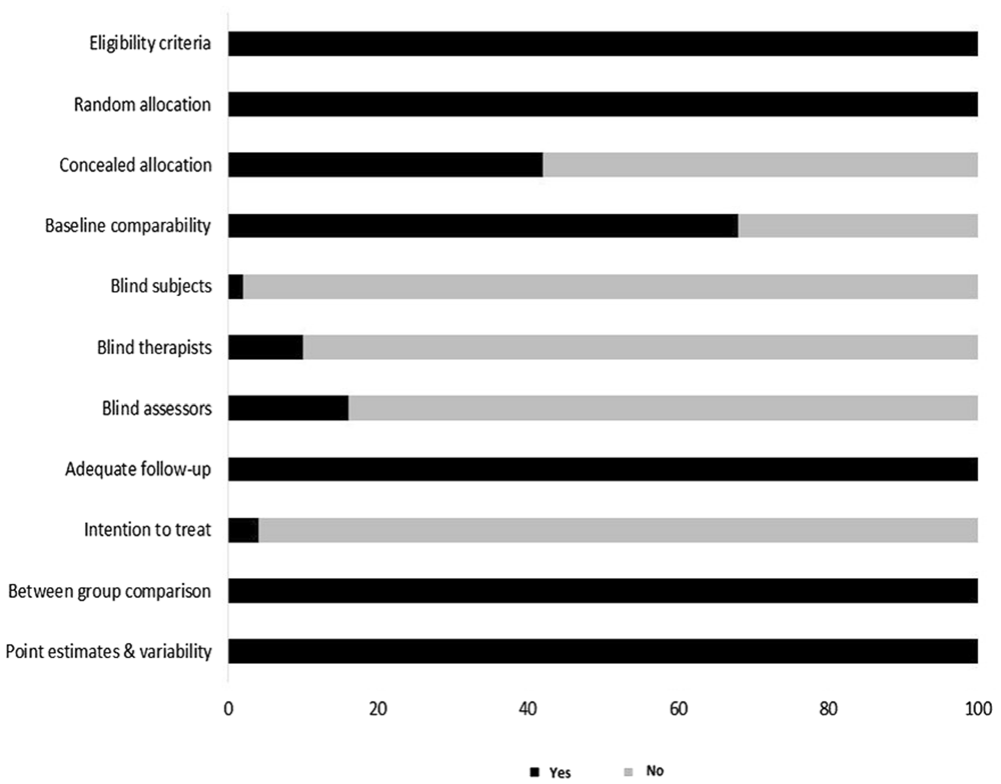
Risk of bias across studies.

To reduce the risks of bias, studies scoring ≥4 on PEDro were included in the review. Moreover, the limitation of research protocols to be included in the review was limited to gold standard randomized controlled trials, cluster randomized controlled trials and controlled clinical trials. The individual scores attained by the studies using the PEDro scale have been reported (Supplementary Tables 1 and 2). The average PEDro score for the fifty included studies was computed to be 5.4 out of 10, indicating fair-quality of the overall studies. Seven studies scored 8, three scored 7, twelve studies scored 6, thirteen studies scored 5, and seventeen studies scored 4. Publication bias was analyzed by plotting a Hedge’s g against standard error (Fig. 2). Asymmetries concerning mean in the funnel plot might suggest bias (either positive or negative). Risk of bias across the studies has been demonstrated in (Fig. 3).

### Meta-Analysis

#### Outcomes

The results suggest clear evidence for a positive impact of rhythmic auditory cueing on spatiotemporal gait parameters amongst parkinsonian patients. An enhancement in gait parameters were also observed when rhythmic auditory cueing was introduced with biological variability^94,95^, and music^56,93,96–98^. In the included fifty studies, one study reported enhancements (p > 0.05)^96^, two studies reported negligible effects^100,101^, one study reported significant reduction of rhythmic auditory cueing on spatiotemporal gait parameters^102^. Forty-six studies reported significant enhancements in primary spatiotemporal gait parameters while receiving rhythmic auditory cueing.

### Meta-analysis report

The evaluation of research studies via meta-analysis requires strict inclusion criteria to efficiently limit the heterogeneity^103^. However, among the pooled group of studies post strict inclusion criteria, some amount of unexplained heterogeneity was still observed. Thereafter, sub-group analyses were performed among homogenous studies to exclude and evaluate the cause of heterogeneity. The evaluated parameters were the spatio-temporal gait parameters such as, cadence, stride length, gait velocity, double limb support duration, and turn time. Analyses were also conducted to evaluate the effects of dual-task conditions, the effects of different training durations, presence/lack of medication, early/late phase of treatment, presence of treadmill, and different tempi at which rhythmic auditory cueing was provided on gait parameters. We included a generalized group analysis combined for all the pooled studies. A separate analysis in addition to clinical controlled trials was performed for high quality randomized controlled trails, for allowing a better interpretation of the direction and magnitude of effects. The main reason for not including the statistical approach within the studies was due to major differences in between assessment methods, patient characteristics, auditory stimuli and lack of descriptive statistics within the manuscript. However, attempts were made to retrieve data from respective corresponding co-authors.

### Gait velocity

Gait velocity was analyzed among thirty-five studies. Additional sub-group data were extracted from thirteen included studies^71,86,87,89,94,99,104–108^. In the additional analyses, two studies analyzed early and late treatment groups^89,99^. Two studies analyzed normal and treadmill gait performance^82,103^. Six studies compared cueing between fast and slow tempo^86,93,104,105,109,110^. Five studies analyzed the effects with only fast paced^111–115^, and two with only slow paced tempo^116,117^. Seven studies analyzed cueing at slow tempo^86,93,104,105,109,116,117^. The fast/slow tempo in the included studies was determined by keeping the patient’s preferred cadence as reference. Three studies analyzed patients in “on” and “off” stages of medications^71,107,110^, signifying the presence and absence of medications, respectively. A positive effect here refers to enhancement in gait velocity, and a negative effect refers to reduction in gait velocity.

**Figure 4:**
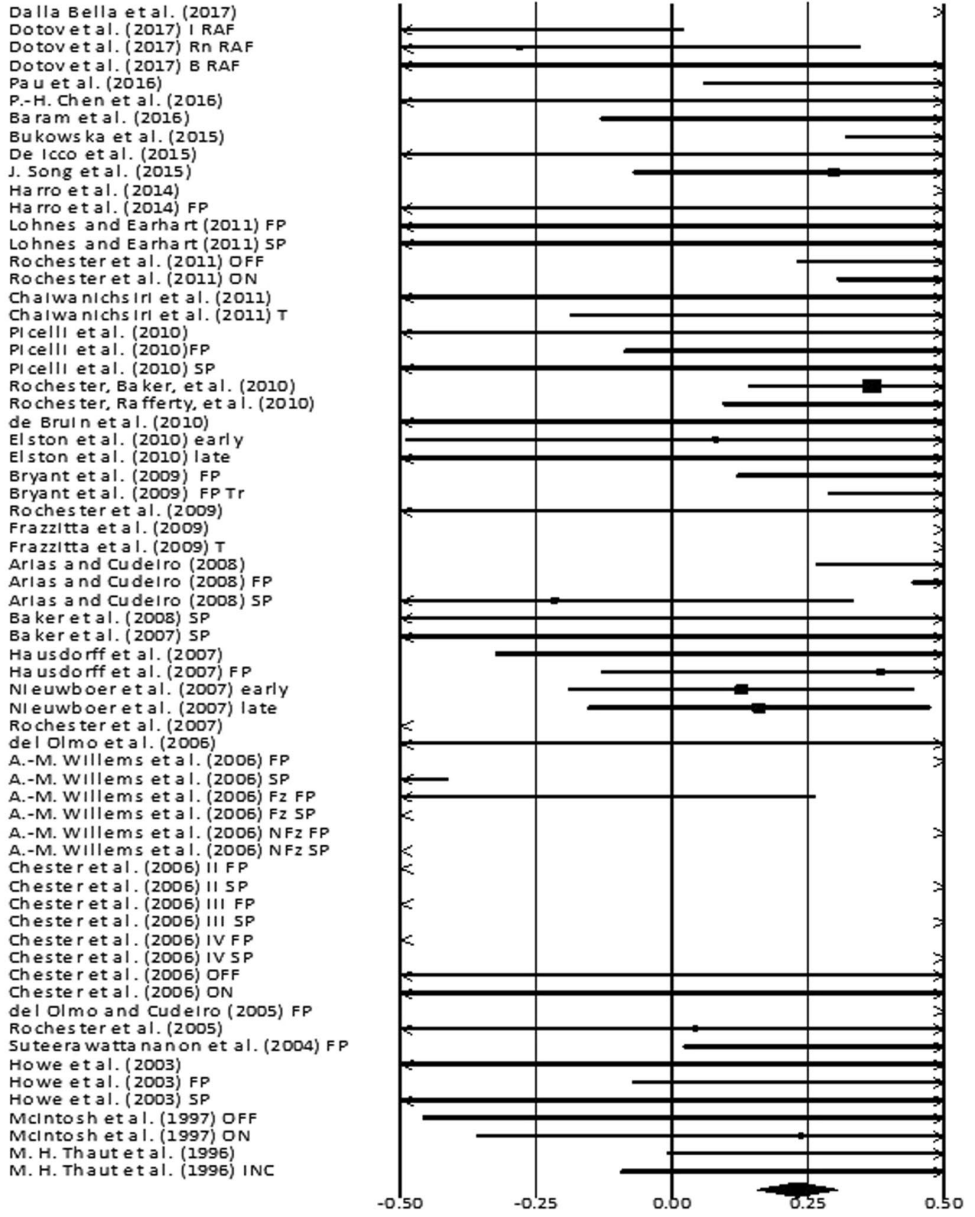
Forest plot illustrating individual studies evaluating the effects of rhythmic auditory cueing on gait velocity among parkinsonian patients. Weighted effect sizes; Hedge’s g (boxes) and 95% C.I (whiskers) are presented, demonstrating repositioning errors for individual studies. The (Diamond) represents pooled effect sizes and 95% CI. A negative effect size indicated reduction in gait velocity; a positive effect size indicated enhancement in gait velocity. (FP: Fast paced, SP: Slow paced, Fz: Freezers, NFz: Non-Freezers, ON: with medications, OFF: without medications, INC: Inclined training, T: Treadmill training, FP T: Fast paced training, I: Isosynchrounous cueing, Rn: Random, BL Biological variability, RAC: Rhythmic auditory cueing).

The analysis of studies revealed (Fig. 4) a *small* effect size in the positive domain (g: 0.23, 95% C.I: 0.1 to 0.3). Substantial heterogeneity was observed in between the studies (I^2^: 87.4%, p > 0.01). Further, sub-group analyses were conducted among homogenous studies to explore heterogeneity. An analysis between “on” and “off” medications patients (Supplementary Figures 1 and 2), revealed a positive *small* effect size for “off” group with negligible heterogeneity (g: 0.43, 95% C.I: 0.11 to 0.75, I^2^: 18.8%, p = 0.29), and positive *medium* effect size for “on” group with negligible heterogeneity (g: 0.55, 95% C.I: 0.23 to 0.87, I^2^: 0.0%, p = 0.44). A sub-group analysis for treadmill training groups (Supplementary Figure 3) revealed a positive *large* effect size with negligible heterogeneity (g: 1.0, 95% C.I: 0.33 to 1.67, I^2^: 24.6%, p = 0.24). A sub group analysis between “fast” and “slow” externally paced auditory cueing (Supplementary Figure 4), revealed a positive *medium* effect for the “fast” group with negligible heterogeneity (g: 0.7, 95% C.I: 0.50 to 0.89, I^2^: 0.0%, p = 0.44), and a negative *small* effect for the “slow” group (Supplementary Figure 5) with negligible heterogeneity (g: −0.24, 95% C.I: 10.51 to 0.19, I^2^: 23.53%, p = 0.24). Further, twenty-one studies analyzing the effects of a simple rhythmic auditory cueing were analyzed (Supplementary Figure 6). The sub-analysis revealed a positive *small* effect size (g: 0.05, 95% C.I: −0.07 to 0.17, I^2^: 86.4%, p < 0.01) with substantial heterogeneity. The analysis revealed two main types of sub-groups analyzing the effects of rhythmic auditory cueing with and without training. The analysis of ten studies analysing the direct effects of rhythmic auditory cueing i.e. without training (Supplementary Figure 7) revealed a negative *small* effect size (g: −0.34, 95% C.I: −0.5 to −0.18, I^2^: 85.9%, p < 0.01) with substantial heterogeneity. The studies were then categorized according to the disease duration of parkinsonian patients in the studies i.e. >9 years or <9 years. Six studies evaluated the effects of rhythmic auditory cueing on gait performance, with patients having mean disease duration <9 years. The analysis revealed a positive *small* effect size (g: 0.16, 95% C.I: −0.12 to 0.44, I^2^: 0%, p = 0.56) with negligible heterogeneity. The studies analysing severe parkinsonian patients i.e. >9 years of disease duration revealed a negative *small* effect size (g: −0.37, 95% C.I: −0.62 to −0.13, I^2^: 91%, p < 0.01) with substantial heterogeneity. Upon further evaluation of heterogeneity in the sub-group we observed that the experimental procedures differed considerably between each other. For instance, Chen *et al*.^118^ analysed gait performance during gait turning, Arias and Cudeiro^104^ utilized a varied range of frequency that differed from other studies, and Rochester *et al*.^119^ utilized a complex functional task that required the patients to perform a sitting to stand and carrying a tray. Therefore, a further sub-analysis was not carried out.

A sub-group analysis for thirteen studies analysing the effects of training with rhythmic auditory cueing (Supplementary Figure 8), revealed a positive *medium* effect size (g: 0.63, 95% C.I: 0.49 to 0.76, I^2^: 74.1%, p < 0.01) with substantial heterogeneity. Further, upon sub-group analysis to reveal the cause of heterogeneity we excluded Frazzitta *et al*.^106^ because the patients in their study had on average a longer disease duration (13.2 ± 4.1 years) than the patients from other studies. Additionally, Dalla Bella *et al*.^99^ was excluded from further analysis. The study’s training regime differed from the other studies i.e. rhythmic auditory cueing with ±10% modulation of pace according to preferred cadence. Moreover, the training included a hand tapping task concurrently with gait training. The analysis after excluding these studies revealed a positive *medium* effect size with moderate heterogeneity (g: 0.64, 95% C.I: 0.37 to 0.92, I^2^: 36.08%, p = 0.34). Moreover, an additional analysis of training duration was performed (more or less than 45 min). The analysis for thirteen studies included a treatment duration for less than 45 minutes (Supplementary Figure 9) revealed a positive *medium* effect size with substantial heterogeneity (g: 0.61, 95% C.I: 0.48 to 0.74, I^2^: 69.3%, p < 0.01). Further, exclusion of Chaiwanichsiri *et al*.^85^, Frazzitta *et al*.^106^ was done as both the studies incorporated treadmill training and a training duration of 20 minutes, and Harro *et al*.^113^ as the authors only included only one training session per week, whilst the others included more than 3 sessions per week. The analysis for 30–45 min of duration (Supplementary Figure 10), revealed a positive *medium* effect size with moderate heterogeneity (g: 0.52, 95% C.I: 0.38 to 0.66, I^2^: 33.8%, p > 0.05). The analysis for 20 min of duration (Supplementary Figure 11), revealed a positive *large* effect size with substantial heterogeneity (g: 1.09, 95% C.I: 0.7 to 1.47, I^2^: 80.9%, p < 0.01). The studies however differed considerably from one another as, Frazzitta *et al*.^106^ included parkinsonian patients in advanced stage of disease, as compared Chaiwanichsiri *et al*.^85^ where patients were in early stages. The two studies analyzing the effects of training in ling duration i.e. more than 45 minutes were considerably different as del Olmo and Cudeiro^112^ utilized rhythmic auditory cueing with an instruction to perform faster gait, and while carrying out a manual task, whereas del Olmo *et al*.^102^ did not incorporate such technique. A sub-group analysis based on the number of weeks the patients received treatment i.e. less or more than 5 weeks was performed. Analysis for patients receiving treatment for less than 5 weeks (Supplementary Figure 12) revealed a positive *medium* effect size with substantial heterogeneity (g: 0.73, 95% C.I: 0.31 to 1.14, I^2^: 21.3%, p > 0.05). Likewise, for patients receiving treatment for more than 5 weeks (Supplementary Figure 13) revealed a positive *small* effect size with negligible heterogeneity (g: 0.46, 95% C.I: 0.2 to 0.72, I^2^: 0%, p > 0.05).

#### Randomized controlled trials

A sub-group analysis on the included randomized controlled trials was performed (Supplementary Figure 14). Two studies analyzed early and late intervention groups^96,117^. Three studies involved a training regime with rhythmic auditory cueing^88,98,113^. One study analyzed immediate effects of rhythmic auditory cueing on gait^121^. The analysis revealed a positive *small* effect for the group with substantial heterogeneity (g: 0.25, 95% C.I: 0.11 to 0.40, I^2^: 73.5%, p = 0.001).

A sub-group analysis between “early” and “late” treatment groups revealed a positive *small* effect size for “early” group (Supplementary Figure 15) with negligible heterogeneity (g: 0.11, 95% C.I: −0.16 to 0.39, I^2^: 0.0%, p = 0.88), and similar *small* effect size for “late” group (Supplementary Figure 16) with negligible heterogeneity (g: 0.11, 95% C.I: −0.16 to 0.39, I^2^: 0.0%, p = 0. 45). A sub-group analysis between de Bruin *et al*.^98^ and Harro *et al*.^110^ revealed a positive *large* effect size with substantial heterogeneity (g: 0.97, 95% C.I: 0.29 to 1.66, I^2^: 93.35%, p < 0.01). The training program differed between the studies, de Bruin *et al*.^98^ trained their patients for at least 3 sessions per week, whereas Harro *et al*.^113^ performed only one training session per week. Gait velocity under dual-task condition was analyzed amongst nine studies. The specifics of dual-tasks have been mentioned (Supplementary Table 1). The analysis (Supplementary Figure 17) revealed a positive *small* effect size (g: 0.38, 95% C.I: 0.09 to 0.66, I^2^: 9.95%, p > 0.05) with negligible heterogeneity.

### Stride length

**Figure 5:**
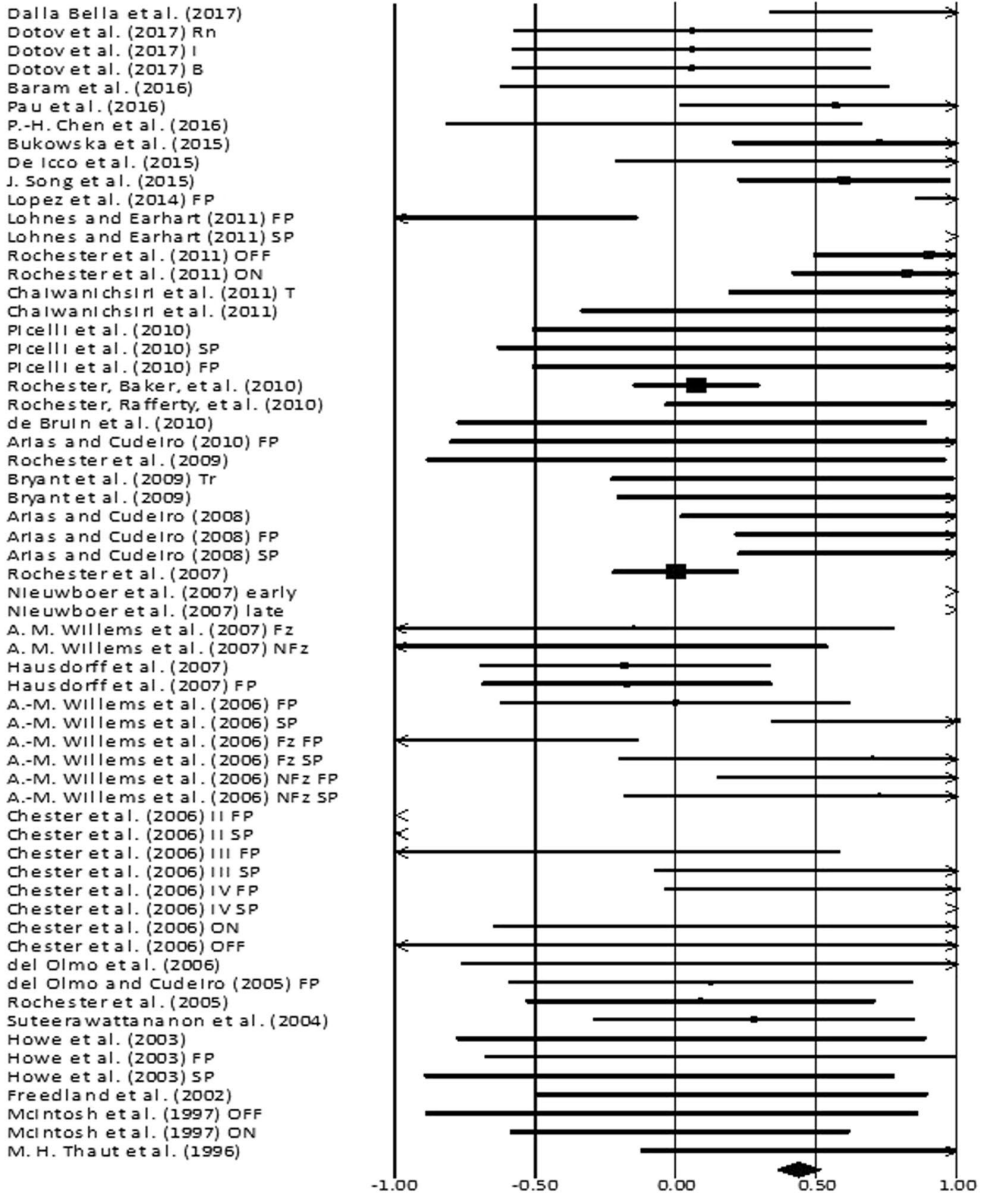
Forest plot illustrating individual studies evaluating the effects of rhythmic auditory cueing on stride length among parkinsonian patients. Weighted effect sizes; Hedge’s g (boxes) and 95% C.I (whiskers) are presented, demonstrating repositioning errors for individual studies. The (Diamond) represents pooled effect sizes and 95% CI. A negative effect size indicated reduction in stride length; a positive effect size indicated enhancement in stride length. (FP: Fast paced, SP: Slow paced, Fz: Freezers, NFz: Non-Freezers, ON: with medications, OFF: without medications, INC: Inclined training, T: Treadmill training, FP T: Fast paced training, I: Isosynchrounous cueing, Rn: Random, BL Biological variability, RAC: Rhythmic auditory cueing).

Stride length was analyzed amongst thirty-four studies. Additional sub-group data was extracted from fourteen included studies. A positive effect here refers to enhancement in stride length, and a negative effect refers to reduction in stride length. The combined analysis revealed (Fig. 5) a positive *small* effect size (g: 0.42, 95% C.I: 0.35 to 0.5, I^2^: 85.05%, p < 0.01) with substantial heterogeneity. A sub-group analysis in between “off” and “on” medication groups was performed among three studies^71,107,110^. The analysis for “on” group (Supplementary Figure 18), revealed a *large* effect size in positive domain (g: 0.77, 95% C.I: 0.45 to 1.1, I^2^: 43.6%, p = 0.16) with moderate heterogeneity. Likewise, analysis for “off” group (Supplementary Figure 19), revealed a *large* effect size in positive domain (g: 0.85, 95% C.I: 0.49 to 1.2, I^2^: 51%, p = 0.12) with marginally moderate heterogeneity. This heterogeneity could possibly be attributed to Chester *et al*.^110^, as the authors utilized a different tempo for rhythmic auditory cueing as compared to the other two counterparts. Post exclusion the meta-analysis revealed *large* effect size for both “on” and “off” in positive domain (g: 0.86, 95% C.I: 0.52 to 1.2, I^2^: 0%, p = 0.64), (g: 0.96, 95% C.I: 0.59 to 1.34, I^2^: 0%, p = 0.39), with negligible heterogeneity, respectively.

**Figure 6:**
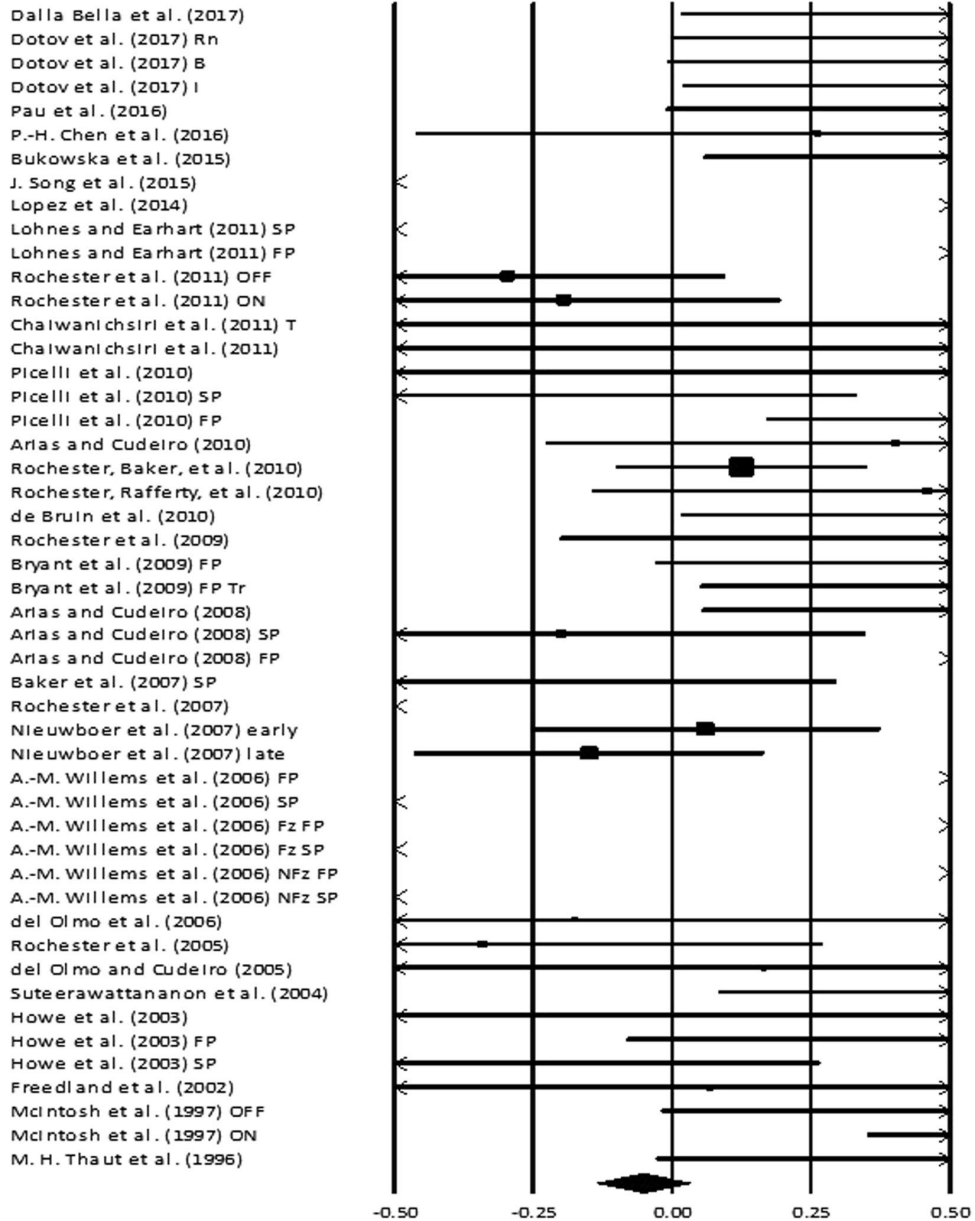
Forest plot illustrating individual studies evaluating the effects of rhythmic auditory cueing on cadence among parkinsonian patients. Weighted effect sizes; Hedge’s g (boxes) and 95% C.I (whiskers) are presented, demonstrating repositioning errors for individual studies. The (Diamond) represents pooled effect sizes and 95% CI. A negative effect size indicated reduction in step frequency; a positive effect size indicated enhancement in step frequency. (FP: Fast paced, SP: Slow paced, Fz: Freezers, NFz: Non-Freezers, ON: with medications, OFF: without medications, INC: Inclined training, T: Treadmill training, FP T: Fast paced training, I: Isosynchrounous cueing, Rn: Random, BL Biological variability, RAC: Rhythmic auditory cueing, step frequency: number of steps/minute).

A further analysis differentiated fast and slow-paced stimuli with respect to the patient’s preferred cadence. Seven studies compared the effects between fast and slow paced rhythmic cueing^86,93,104,105,109–111^, whereas four studies analyzed the effects of only fast paced cueing^31,91,112,114^. Analysis for fast-paced stimuli among eleven studies (Supplementary Figure 20), revealed a positive *small* effect size (g: 0.27, 95% C.I: 0.09 to 0.4, I^2^: 69%, p < 0.01) with substantial heterogeneity. Chester *et al*.^110^ was excluded from further analysis as the authors evaluated the effects of fast and slow-paced stimuli on patients with different stages of severity. Likewise, Lopez *et al*.^91^ utilized a tempo faster than the other studies i.e. +25% of preferred cadence, hence was also excluded. Thereafter, a positive *small* effect size (g: 0.3, 95% C.I: 0.08 to 0.52, I^2^: 0%, p = 0.7) with negligible heterogeneity was observed. Similarly, for the slow-paced stimuli five studies were evaluated (Supplementary Figure 21) a positive *small* effect size (g: 0.43, 95% C.I: 0.18 to 0.69, I^2^: 83.2%, p < 0.01) with substantial heterogeneity was observed. After excluding (Chester *et al*., 2006) we observed a positive *medium* effect size (g: 0.69, 95% C.I: 0.35 to 1.03, I^2^: 20.03%, p = 0.29) with negligible heterogeneity. Studies analyzing only the effects of un-modulated rhythmic auditory cueing were analyzed on twenty-three studies (Supplementary Figure 22) and they revealed a positive *small* effect size (g: 0.35, 95% C.I: 0.22 to 0.48, I^2^: 35.3%, p = 0.04) with moderate heterogeneity. Studies were then separated based on training or direct application of rhythmic auditory cueing. A sub-group analysis for nine studies analyzing direct application of rhythmic auditory cueing among nine studies (Supplementary Figure 23), revealed a positive *small* effect size (g: 0.12, 95% C.I: −0.11 to 0.35, I^2^: 0%, p = 0.72) with negligible heterogeneity. Moreover, for the training group thirteen studies (Supplementary Figure 24) were evaluated and the analysis revealed a positive *small* effect size (g: 0.37, 95% C.I: 0.23 to 0.51, I^2^: 26.2%, p = 0.16) with moderate heterogeneity. For training group with 30 minutes of training (Supplementary Figure 25), the analysis revealed a positive *small* effect size (g: 0.36, 95% C.I: 0.21 to 0.51, I^2^: 42.5%, p = 0.07) with moderate heterogeneity. Three studies with different training regimes i.e. 20 minutes^85^, and 1 hour duration were excluded^102,112^. A further analysis determining treatment duration across more than or less than 5 weeks revealed *medium* positive effect size (g: 0.61, 95% C.I: 0.44 to 0.78, I^2^: 71.2%, p > 0.1) with substantial heterogeneity. Further sub-group analysis for less than 5 session per week of training revealed *small* positive effect size (g: 0.39, 95% C.I: 0.08 to 0.7, I^2^: 0%, p < 0.11) with negligible heterogeneity^122,123^. For studies analyzing training for more than 5 sessions per week (Supplementary Figure 26), revealed *small* positive effect size (g: 0.4, 95% C.I: 0.1 to 0.68, I^2^: 0%, p > 0.5) with negligible heterogeneity.

#### Randomized controlled trials

A sub group analysis for 4 randomized controlled trials (Supplementary Figure 27) revealed a positive *medium* effect size (g: 0.56, 95% C.I: 0.42 to 0.69, I^2^: 98.04%, p < 0.01) with substantial heterogeneity. A sub group analysis for two randomized controlled trials analyzing the effects of rhythmic auditory cueing without training revealed no effect (g: 0.0, 95% C.I: −0.28 to 0.3, I^2^: 0%, p = 0.9) with negligible heterogeneity. Stride length under dual-task condition was analyzed amongst eight studies (Supplementary Figure 28). The analysis revealed a positive *small* effect size (g: 0.31, 95% C.I: 0.14 to 0.48, I^2^: 0%, p = 0.8) with negligible heterogeneity.

### Cadence

Cadence was analyzed amongst thirty studies (Figure 6). Additional sub-group data was extracted from eleven included studies. The analysis of studies revealed a negative *small* effect size (g: −0.05, 95% C.I: −0.13 to 0.03, I^2^: 93.6%, p < 0.01) with substantial heterogeneity. A positive effect here refers to enhancement in step frequency i.e. number of steps per minute, and a negative effect refers to reduction in step frequency.

Two studies compared the effects of “on” and “off” phase of medications on the patient's affected from parkinsonism^71,107^. A sub-group analysis for “off” treatment groups (Supplementary Figure 29) revealed a negative *small* effect size (g: −0.1, 95% C.I: −0.46 to −0.25, I^2^: 81.97%, p = 0.01) with substantial heterogeneity. Likewise, for “on” treatment group (Supplementary Figure 30), a positive *small* effect size (g: −0.13, 95% C.I: −0.20 to 0.46, I^2^: 89.69%, p < 0.01) with substantial heterogeneity was observed. The heterogeneity could be attributed to the use of different tempi i.e. at preferred cadence, at tempo faster than preferred cadence, during rhythmic auditory cueing training by McIntosh *et al*.^107^. Moreover, sub-groups analyses were performed for gait performance with “fast” and “slow” paced tempi with respect to patient’s preferred cadence. Five studies compared effects of fast and slow paced stimuli^86,93,104,105,109^, whereas two studies evaluated the effects of only fast paced stimuli on gait performance^111,115^. Further, seven studies analyzed only fast-paced stimuli (Supplementary Figure 31), a positive *large* effect size (g: 1.0, 95% C.I: 0.78 to 1.34, I^2^: 87.05%, p < 0.01) with substantial heterogeneity was observed. A sub-group analysis lead to exclusion of three studies based on the severity of patients included i.e. >9 years of disease duration^85,104,109^. The analysis of fast-paced stimuli in less severe patients revealed a positive *medium* effect size (g: 0.61, 95% C.I: 0.25 to 0.94, I^2^: 0%, p = 0.49) with negligible heterogeneity. In severe patients, we observed a positive *large* effect size (g: 1.75, 95% C.I: 1.31 to 2.18, I^2^: 74.6%, p = 0.01) with substantial heterogeneity. However, we excluded Willems *et al*.^86^, from further analysis as the authors incorporated a faster tempo i.e. + 20% as compared to + 10% in the other studies^104,109^. We observed a positive *large* effect size (g 1.4, 95% C.I: 0.89 to 1.91, I^2^: 27.6%, p = 0.24) with marginally moderate heterogeneity. Six studies analyzed the effects of slow-paced stimuli on gait performance in patients (Supplementary Figure 32). We observed a negative *large* effect (g −1.25, 95% C.I: −1.59 to −0.92, I^2^: 89.34%, p < 0.01) with substantial heterogeneity. Further, on dividing the studies into two categories i.e. >9 years of disease duration (severe and less severe). We observed a negative *medium* effect (g −0.5, 95% C.I: −0.97 to −0.04, I^2^: 0%, p = 0.93) with negligible heterogeneity, in less severe group. Whereas, a negative *large* effect (g −2.05, 95% C.I: −2.53 to −1.57, I^2^: 92.45%, p < 0.01) with substantial heterogeneity was observed in the more severe group. Further, we excluded Willems *et al*.^86^ because the patients differed considerably in terms of age, disease duration and treatment. We then observed a negative *large* effect (g −1.54, 95% C.I: −2.06 to −1.02, I^2^: 0%, p = 0.37) with negligible heterogeneity. Twenty studies analyzing rhythmic auditory cueing at preferred cadence revealed a positive *small* effect size (g 0.17, 95% C.I: 0.01 to 0.32, I^2^: 90.5%, p < 0.01) with substantial heterogeneity (Supplementary Figure 33). On further sub-group analysis nine studies analyzing only rhythmic auditory cueing (Supplementary Figure 34), implementation without training revealed a positive *small* effect size (g 0.30, 95% C.I: 0.07 to 0.53, I^2^: 0%, p = 0.45) with negligible heterogeneity. Thereafter, studies analyzing only rhythmic auditory cueing implementation with training (Supplementary Figure 35), revealed a *small* negative effect size (g: 0.04, 95% C.I: −0.1 to 0.2, I^2^: 93.6%, p < 0.01) with substantial heterogeneity. Studies analyzing only rhythmic auditory cueing implementation with 30 min (Supplementary Figure 36), of training revealed a *small* effect size (g: 0.09, 95% C.I: −0.06 to 0.25, I^2^: 95.5%, p < 0.01) with substantial heterogeneity. Further, we excluded Song and Ryu^124^, Pau *et al*.^122^, and de Bruin *et al*.^98^ as the authors included the training for 8, 12 and 13 weeks, respectively. Furthermore, two studies differing considerably in training regimes were excluded from further analysis^88,125^. Studies analyzing only rhythmic auditory cueing implementation with training for a few sessions in less than 5 weeks (Supplementary Figure 37), revealed a positive *medium* effect size (g: 0.65, 95% C.I: 0.33 to 0.96, I^2^: 0%, p = 0.94) with negligible heterogeneity. An analysis of studies evaluating rhythmic auditory cueing with training more than 5 days per week (Supplementary Figure 38) revealed a negative *small* effect size (g: −0.22, 95% C.I: −1.16 to 0.71, I^2^: 23.6%, p > 0.05) with negligible heterogeneity.

### Randomized controlled trials

We analyzed three randomized controlled trials which evaluated the effects of rhythmic auditory cueing on cadence (Supplementary Figure 39). Upon analysis, we observed a *small* effect (g 0.07, 95% C.I: −0.08 to 0.22, I^2^: 44.6%, p = 0.13) with moderate heterogeneity. Cadence under dual-task condition was analyzed amongst nine studies (Supplementary Figure 42). The analysis revealed a positive *small* effect size (g: 0.11, 95% C.I: −0.06 to 0.8, I^2^: 0%, p = 0.8) with negligible heterogeneity.

### Double limb support phase

Double limb support phase was analyzed amongst eight studies^56,86,110,111,114,120,122,126^. Additional sub-group data was extracted from three included studies^86,89,110^. A positive effect here refers to increase in total duration when both feet are in contact with the ground, and vice versa for the negative effect. The analysis (Supplementary Figure 43) revealed a positive *medium* effect size (g: 0.5, 95% C.I: 0.34 to 0.67, I^2^: 93.46%, p < 0.01) with substantial heterogeneity. With a fast-paced stimulus the rhythmic auditory cueing (Supplementary Figure 44), reveals a *small* effect size in positive domain (g: 0.46, 95% C.I: 0.05 to 0.87, I^2^: 92.3%, p < 0.01) with negligible heterogeneity. Two studies with slow-paced stimuli (Supplementary Figure 45) yielded *small* positive effects (g: 0.33, 95% C.I: −0.18 to 0.85, I^2^: 92.8%, p < 0.01) with substantial heterogeneity. This heterogeneity could possibly be attributed to the range of severity i.e. stage II, III and IV of parkinsonism in^110^. Finally, analysis with rhythmic auditory cueing at preferred cadence (Supplementary Figure 46), revealed reduction in double limb support phase with *medium* effect size in negative domain (g: −0.56, 95% C.I: −0.9 to −0.22, I^2^: 0%, p = 0.72) with negligible heterogeneity.

### Turn time

Three studies analyzed the effects of rhythmic auditory cueing on turn time^86,110,111^. A positive effect here refers to increase in total duration for performing a turn during gait, and vice versa for the negative effect. The analysis revealed a negative *large* effect size (g: −2.2, 95% C.I: −2.49 to −1.94, I^2^: 83.8%, p < 0.01) with substantial heterogeneity. Arias and Cudeiro^31^ were excluded from further analysis as the authors utilized a rhythmic auditory cueing with faster tempo. The studies were then segregated according to their patient’s characteristics as freezers and non-freezers. The meta-analysis for freezers revealed negative *large* effect size (g: −2.08, 95% C.I: −2.5 to −1.66, I^2^: 93.7%, p < 0.01) with substantial heterogeneity. Further, an analysis for non-freezers revealed negative *large* effect size (g: −2.3, 95% C.I: −2.71 to −1.88, I^2^: 87.67%, p < 0.01) with substantial heterogeneity. The heterogeneity cannot be further explained here.

## Discussion

The primary objective of this present systematic review and meta-analysis was to develop a current state of knowledge for the effects of rhythmic auditory cueing on gait stability in parkinsonian patients. Out of fifty-included studies 88% studies reported beneficial effects of rhythmic auditory cueing on gait parameters. Further, the meta-analysis yielded significant small-to-large standardized effects for the benefits of rhythmic auditory cueing on spatiotemporal gait parameters for parkinsonian patients. Previous studies have reported substantial negative effects of parkinsonism on spatial parameters of gait for instance, stride length, and gait velocity. The current analysis revealed that both stride length (g: 0.48) and gait velocity (g: 0.27) can be enhanced by rhythmic auditory cueing. However, a generalized negative effect of rhythmic auditory cueing was observed on cadence (g: −0.13). Generally, patients with parkinsonism are characterized with reduced gait velocity, stride length, foot clearance, increased cadence, narrowed base of support, festination and in advanced cases freezing of gait^127,128^. The primary underlying physiological reason being inability to generate a substantial amplitude of motor movements^128^, possibly due to deficits in internal timing of movements^45,129–131^.

From a neurophysiological aspect, Spaulding *et al*.^43^ suggested discrepancies in sensory-motor interactions which might lead to such autonomic disruptions. Nombela *et al*.^45^ reattributed and mentioned the dysfunction of an internal cueing system which is associated with coordinating a information exchange between basal ganglia and supplementary motor area. Moreover, studies have also suggested degeneration of a widespread neural network in Parkinson’s disease including cerebellum, basal ganglia, somatosensory area and pre-somatosensory area during the degenerative process^45,131^. Kotz and Schwartze^132^ reported that during the preclinical stage, hyperactivity in pre-somatosensory area might be a compensatory mechanism for cerebellar dysfunctions. Likewise, in advanced stages selective loss of pyramidal neurons in pre-somatosensory area might result in its underactivity, followed by deficits in temporal processing^45,132^, possibly leading to motor block or freezing instances during gait.

The use of rhythmic auditory cueing has been discussed widely in published literature^20,43,45,53,60,74^. This medium of entrainment transfer has been speculated to bypass the affected basal ganglia network (pallidal-supplementary motor area) via another alternative pathway^114,133,134^. Moreover, Fujioka *et al*.^65^ reported modulation of neuromagnetic β oscillations with rhythmic auditory stimuli in auditory cortex, cerebellum, inferior frontal gyrus, somatosensory area and sensorimotor cortex. The stimuli has been suggested to activate inferior colliculi^135^, cerebellum, brainstem^114,136^, sensorimotor cortex^137,138^, further instigating reorganization in cortico-cerebellar circuits^70^. Rhythmic auditory cueing has also been suggested to reap the benefits of the preserved neural centres^139^, involved in perceiving externally cued and goal directed movements amongst parkinsonian patients (see also “kinesia paradoxica”^140^). The authors proposed that motor activities directed by external sensory cueing evoke pathways via cortical, premotor areas^141^, effectively bypassing the affected basal ganglia region^95^. Studies have suggested that rhythmic sensory cues can also replace deficient pallidal-cortical projections, activate the supplementary motor area and aid in motor tasks by mimicking feedforward input, thereby reducing bradykinesia, and associated motor deficits^142^. Similarly, the external cueing can supplement critical spatio-temporal information which is necessary for initiation or facilitating motor activities^30,89^, such as during gait or arm movements^69,143^. In context of gait execution the external rhythm can guide the patients to synchronize their ground contact and lift-off times^144^. The auditory patterns might also assist the planning of a motor command before executing a movement^145^. Moreover, the periodicity in rhythmic auditory cueing has also demonstrated to effectively reduce variability in musculoskeletal activation patterns, thereby allowing more economical and consistent motor unit recruitment^46^, further smoothing the velocity and acceleration profiles of joint motions by scaling movement time^46^.

Typical pharmacological interventions for controlling motor symptoms in parkinsonism include levodopa, dopamine agonists and monoamine oxidase type B inhibitors^40^. Rochester *et al*.^71^ interestingly mentioned the limitations of dopaminergic medications on gait dysfunctions associated with degeneration of non-dopaminergic pathways^88^. The medications allow only symptomatic relief and offer no relief from the underlying pathology^146^. Moreover, their benefits in terms of enhancement of gait performance is still debatable. Benefits in turn time^147^, stride length, gait speed^148^, have been reported in some studies. While some studies report no effects on gait speed^149^, cadence^150^, stride time variability^151^, double limb support duration^152^, and reduction in postural stability^147^. The current meta-analysis observed beneficial effects of concurrent application of medications and rhythmic auditory cueing. The analyses reported marginally larger effect sizes for stride length (g: 0.96) and gait velocity (g: 0.55) during the “on” phase of medications, in comparison to the “off” medication group for stride length (g: 0.86) and gait velocity (0.43). However, such differences were not found for cadence, where small negative effect sizes were observed in both “on” (−0.13) and “off” (−0.10) conditions. It is important to note that this analysis shows the compensatory role of rhythmic auditory cueing for counteracting motor deficits in the absence of medications. Although, studies have reported the benefits of the medications in short-term^40^, a long term cost concerning motor dysfunction has also been reported^146^. Long-term consumption of medications i.e. both levodopa and levodopa sparring therapy has been associated with severe consequences on health and quality of life such as dyskinesis, loss of drug efficacy and toxicity^40,146^. This is possibly due to levodopa associated decline in dopamine transported integrity located in nigrostriatal nerve terminals^153^. Likewise, the progression of disease has shown to reduce the effectiveness of medications^154^, especially on gait characteristics^148^. Therefore, the findings in the present review strongly suggest the use of rhythmic auditory cueing as an adjunct therapy with medications to curb the motor deficits in Parkinson’s disease. Moreover, we suggest future studies to analyse the long-term effects of rhythmic auditory cueing with withdrawal of parkinsonian medications, to observe whether the enhancements obtained are resilient and are retained, or not.

Another crucial factor in rhythmic auditory cueing that might significantly influence the rehabilitation progress of a parkinsonian patient is “change in tempo”. For instance, change in tempo has been associated with various neurophysiological changes such as, increased neuronal activation in fronto-occipital networks^155^, excitability of the spinal motor neurons by reticulospinal pathways, which might possibly reduce the response time for a motor task. Likewise, variation in tempo during training is suggested to be beneficial for maintaining a healthy gait pattern, as constant rhythmic pattern for longer durations have shown to decrease fractal scaling of stride times from healthy 1/f structure, possibly because of organization of stride time variability around a single frequency^156–158^. Additionally, Buchecker *et al*.^159^ demonstrated beneficial effects of enhanced variability within training on posture and electromyographic activity (see more from “dynamic system theory”^160^). This might serve to be beneficial for parkinsonian patients to learn how to regulate gait, when passing through fall-prone environments. Moreover, the induction of variability can also be subjected subliminally (for instance changes in tempo, frequency, timbre, interstimulus interval, see also^161^). This might maintain variability in the rehabilitation protocol and simultaneously prevent any conscious stress to excessively speed up, or slow down the gait. Future studies can elucidate these effects by evaluating variability in both the auditory and environmental components within training paradigms. In the current analyses, our aim was to determine the extent of tempo shift which might be beneficial in a rehabilitation protocol. Previous studies have shown that healthy participants can easily modulate gait parameters to changes such as ±20%^162,163^, however parkinsonian patients have failed to demonstrate such effects^86^. Supposedly, an exceedingly fast tempo might surpass patient’s physiological capabilities and could possibly promote the patient in a high-stress situation^20^. Further this increased tempo associated enhancement in gait velocity, cadence, and double limbs support parameters can lead to a speed-accuracy trade off^86,164^. On the contrary, too slow tempo, for instance might allow the participant more time than required to execute a movement, possibly promoting movement specific re-investment^1,24^. Therefore, the extent of tempo shift should be tailor made according to the patients’ capabilities.

Fast pace stimuli i.e. ≤ +10% has been suggested to effectively counteract reduction in gait velocity, cadence, stride length^109^. We observed enhancement in gait velocity (g: 0.7), cadence (1.0), and stride length (0.30). Likewise, use of fast paced tempo is to be encouraged during the early phase of disease. Willems *et al*.^86^ suggested an association between tempo reduction and enhanced stride length, but also with reduced cadence and gait velocity. This could possibly be attributed to a speed-accuracy trade-off mechanism, where reduction in gait velocity but enhancement in stride length offers slow, but stable gait performance^165^. The present meta-analysis with the application of slow-paced tempo i.e. ≥+ 10% reported benefits in stride length (g: 0.69), reduction in cadence (−1.25), and gait velocity (−0.24). Thereby, suggesting an efficient manoeuvre to counteract the shuffling gait characteristic in parkinsonian patient i.e. short stride length with faster cadence^164,166^, especially during the advanced stages of disease where rehabilitation aims should focus more on mobility with stability. Gait training with rhythmic auditory cueing at preferred cadence also has shown to allow benefits in gait velocity (g: 0.43), stride length (0.6), cadence (0.46), reduction in turn time (−2.2), and double limb support phase (−0.56). However, a regular use of the same tempo at preferred cadence might impact recovery in terms of fractal scaling. Therefore, in terms of practical application of different tempo in rehabilitation protocols we suggest utilization of preferred, slow and fast tempi (±10% of preferred cadence), to maintain variability in gait during training.

As per the training dosage that should possess most beneficial effects, we observed fourteen studies analysing the effects of rhythmic auditory training with 30 minutes duration, two studies each analysed the training for 20, 45 minutes and 1 hour. Beneficial enhancements in gait parameters were observed in all the studies analysing the effects during a 30 minutes gait training session. These effects were also evident during a 45 minutes session, and for 20 minutes sessions. However, one study analysing the effects of training for long sessions (1- hour) revealed beneficial effects with a fast paced stimuli^112^, while the other revealed no effects^112^. We believe, both mental and physical fatigue could have played a crucial aspect for affecting the gait parameters^167^ during the long sessions. Nevertheless, more evidence from training studies is required to ascertain the negative effects of long training sessions. Based on the current evidence we strongly suggest limiting the treatment duration between 25–40 minutes/ session. Likewise, a minimum of at least 3–5 sessions of rehabilitation are suggested per week, because highest enhancement in stride length (g: 0.39), cadence (0.65) and gait velocity (0.73) were observed during this period. However, this analysis of training dosage must be carefully interpreted as substantial heterogeneity was observed within studies, due to difference in severity and training regimes. These suggestions are in line with the findings of Nascimento *et al*.^168^ where the authors reported application of rhythmic auditory cueing for 30 minutes and for 4 times a week for stroke patients.

It is important to note that the retention of enhancements in gait parameters relies not only on the training received in the clinic but also depends largely on how much the patient follows the treatment protocol at home. The patient usually spends limited amount of time in a rehabilitation setting. Therefore, performing and re-executing the tasks effectively and regularly at home is vital for enhancements in motor performance and quality of life^112^. Lim *et al*.^11^ for instance, reported enhancement in walking activity to 35 minutes per day (qualifying the 30 minutes criteria by centres for disease control and prevention^169^) post home-based gait training with rhythmic auditory cueing. In addition, a home-based training device allowed a 4.2% increase in posture and gait score, 5.5% reduction in freezing instances, 4 cm increase in step length and a 5 cm increase in walking speed^120^. This type of home-based intervention could possibly be beneficial for people lacking proper exposure to medical interventions in developing countries^125^. For instance, parkinsonian patients lacking effective treatment can utilize smartphone devices with dynamic metronome apps such as Walkmate^157^, Listenmee^91^, which with proper medical guidance might allow curbing the motor deficits associated with Parkinson’s disease^170^. In addition, combining the use of external rhythmic entrainment process with different treatment strategies might be a useful tool in rehabilitation as Post *et al*.^171^ suggested the most effective rehabilitation protocol for parkinsonian patients to be multidisciplinary. We included studies analysing the beneficial effects of co-joint application of treadmill and rhythmic auditory cueing. Combining treadmill in gait training sessions offered additional benefits as compared to conventional over ground sessions^38^. For instance, Bello *et al*.^172^, reported improvements in stride length, gait speed, time up and go performance and static postural stability, with retention evitable one-month post training. The current meta-analysis revealed beneficial effects of treadmill training in gait velocity (g: 1.0).

Additionally, using the rhythmic entrainment factor with music, could possibly provoke benefits in both psycho-physiological domains^173–177^. For instance, regulating stress levels, mediating arousal, emotions, internal motivation, memory, attention, executive functions^178,179^, muscle power^180^, and endurance^178^. Modifications in the types of auditory cueing can also impart differential effects on psycho-physiological aspects of performance. For instance, timbre of an auditory input at a higher intensity merged in a broad ascending melody and a rich harmony can possibly motivate a patient to exert more power^182,183^. Also, parkinsonian and associated ageing changes in patients often characterize a higher threshold for action relevant acoustic input, therefore using ecologically valid action related sounds convening spatio-temporal information can possibly enhance saliency of sensory information, transferring spatio-temporal information effectively and therefore providing more benefits^94,95,184,185^. This was also demonstrated by Dotov *et al*.^94^, and^95,185^. These authors demonstrated beneficial effects on spatio-temporal gait parameters with biologically variable rhythmic auditory cueing as compared to isosynchronous cueing. Thereby, suggesting potential for modification of auditory signal characteristics for enhancing motor performance in parkinsonian patients. Further methods providing real-time auditory information could possess considerable benefits for enhancing gait performance. One of these methods is movement sonification^73^: here movement parameters are transformed in real-time to sound with an aim to enhance motor perception and performance by targeting areas associated with biological motion perception^66,186,187^. Although few research has been carried out to analyse its effects on parkinsonian gait performance^95,188^, yet, several studies highlight its impact on motor performance and its potential for motor rehabilitation^189,190^. Schmitz and Effenberg^190^ have shown that the synchronization of cyclic movement patterns with movement sonification reduces variability and increases constancy of movements as compared to discrete auditory stimuli. Furthermore, listening to sonified human movements in contrast to or in addition to non-human auditory stimuli seems to influence movement timing and strengthen entrainment effects^191^, possibly by activating mechanisms of biological motion processing in the human brain^66,192^. Moreover, listening to sonification might allow parkinsonian patients to identify their own movement amplitudes and compare their sound with the sound of an auditory movement model, thereby creating a new auditory reference frame. This reference framework might allow a comparison between instructed and intended movement, possibly amplifying the internal representation of movements^193^. This might then induce effects on motor behaviour beyond rhythmic adjustments^58^.

Moreover, counteracting alleviation in conscious attention towards autonomic control, in parkinsonian patients is very critical. Several studies have tried to co-jointly analyze the effects of dual-tasks and rhythmic auditory cueing i.e. to analyze the robustness of auditory-motor coupling with higher information processing constraints^109,116^. Dual-tasks are expected to protect the automaticity of the motor tasks, by possibly engaging information processing resources necessary for conscious control (see also constrained action hypothesis, [27]). This present analysis observed small effects on gait parameters with dual task application i.e. gait velocity (g: 0.38), stride length (0.31), and cadence (0.11). Beneficial effects on age related controls have been reported during similar interventions^109^. Nevertheless, rhythmic auditory cueing both with and without training reduced the constraining effects of a manual dual task over gait^88,121^. Interpretations from our results however suggest that rhythmic auditory cueing counteracts cognitive constraints imposed by cognitively demanding dual-tasks such as carrying a tray, and that this external cueing might be useful in counteracting fall prone situations such as escalators, traffic signals (see more cross-modal overload substitution^194^). Moreover, dual-task training has been suggested to impart beneficial impacts on stability, as the training phase might allow smoothing of cognitive abilities^195^. Possibly, including dual-task training regimes with different complexities with rhythmic auditory cueing might enhance functional rehabilitation progress, self-dependence for instance while carrying out activities of daily living. Lastly, patients with Parkinson’s have been shown to demonstrate considerable rigidity in trunk motions^196^, possibly leading to asymmetry, reduction in arm swing amplitude^197^, and trunk rotation during gait^198^. Son and Kim^199^ reported beneficial effects of rhythmic auditory cueing for increasing arm swing amplitude (36.4° ± 3° vs 25.2° ± 2.8°) and trunk rotation (7° ± 1.3° vs 6.6° ± 0.9°). Thereby, suggesting the beneficial effects of rhythmic auditory cueing beyond the spatiotemporal parameters of gait for enhancing stability.

Our results are consistent with the findings of previous meta-analysis by Spaulding *et al*.^43^, stride length (g: 0.49) and gait velocity (0.54), and cadence (g: 0.55). However, the review did not analyse the quality of included studies, and abstained from performing a heterogeneity analysis. Moreover, Rocha *et al*.^42^ included only seven studies and reported moderate-to-substantial heterogeneity in between studies and abstained from performing sub-group analysis to evaluate the reason for heterogeneity. Therefore, this present literature review for the first time bridges the gap in parkinsonian literature concerning the effects of presence/absence of medications, tempo variations, dual-task settings, and training dosage for improving gait performance with rhythmic auditory cueing.

In conclusion, this review strongly suggests the early incorporation of rhythmic auditory cueing for enhancing gait performance in patients affected from parkinsonism. The results based on meta-analysis suggests training with rhythmic auditory cueing should include tempo variations of ±10% with respect to the preferred cadence, for a minimal period of 20–45 minutes per day, for at least 3–5 days per week. However, in the absence of such facilities as in developing countries, smartphone based apps should be promoted by medical practitioners for home based therapy.

## Electronic supplementary material

Supplementary file
