# Supplementary material for: Effect of rhythmic auditory cueing on parkinsonian gait: A systematic review and meta-analysis
Source: Sci Rep. 2018 Jan 11;8:506. doi: 10.1038/s41598-017-16232-5 (PMC5764963; doi:10.1038/s41598-017-16232-5)
Supplement: Supplementary file 1 — Supplementary file [file 41598_2017_16232_MOESM1_ESM.pdf]

Effect of rhythmic auditory cueing on parkinsonian gait: A systematic review & meta-analysis

Shashank Ghai[1]*, Ishan Ghai[2], Gerd Schmitz[1], Alfred Oliver Effenberg[1]
*Corresponding Author
[1]Institute for Sports Science, Leibniz University Hannover, Germany
[2]School of Life Sciences, Jacobs University Bremen, Germany

Table 1 Studies analysing the effects of rhythmic auditory cueing on gait

Author	Research question(s)/ hypothesis	Sample description, age: (M ± S.D)	PEDro score	Disease duration	Assessment tools	Research design	Sonified elements	Conclusion
Dotov, et al. [91]	Effects of auditory cueing on gait in patients affected from Parkinson's disease	Exp: 7F, 12M (60)<br>Ct: 7F, 12M (60)	6	Exp: 6 (3-20 years)	Coefficient of variation of inter stride interval, cadence, gait velocity, stride length, deterended fluctuation analysis of short-long term series of inter-response-interval correlations, circular statistics for synchronization of footfall and beat	Pre-test, gait performance with/without rhythmic auditory cueing (no variability, biological variability, non-biological variability; randomized), post-test	Rhythmic auditory cueing with no variability, biological variability and non-biological variability at +10% of preferred cadence<br>Magnitude of biological and non-biological variability: 2% of inter-beat-interval<br>Metronome sequence: triangle timbre<br>Musical excerpts<br>Amplitude modulated noise: Modulated on musical excerpt with drum ensemble, discarding tonal information	Significant enhancement in cadence and coefficient of variation for inter stride interval after rhythmic auditory cueing in all conditions for both Exp and Ct.<br>Significant effect of rhythmic auditory cueing that was amplitude modulated for biological variability as compared to isosynchronous metronome cueing on short-long term correlation for term series of inter-response-interval correlations in both Exp and Ct.<br>Enhanced synchronization, but reduced short-long term correlation for term series of inter-response-interval correlations during metronome based isosynchronous cueing as compared to cueing with amplitude modulated for biological variability in both Exp and Ct.
Dalla Bella, et al. [93]	Effects of auditory cueing on gait in patients affected from Parkinsonism	Exp: 5F, 9M (66.5±7.2)<br>Ct: 10F, 10M (66.4±7.8)	6	Exp: 8.0±2.8 years	Gait: Cadence, stride length, stride length variability, gait speed, stride time, stride time variability, synchronization accuracy and inter-step interval<br>Hand tapping: Adaptation Index, phase correction, synchronization accuracy and variability	I: Pre-test, 3 training session/ week for 1 month. Three trials, 30 minutes each with 3 phases (10 minutes each) i.e. 1st and 3rd phase cued with music for 8 min, followed by 2 min of no feedback gait, post-test, follow-up 1 month after training<br>II: Hand-tapping in isochronous sequence of 60 piano tones, same procedure above	Music beat rhythm adjusted to patient's preferred cadence<br>Patients trained with beat frequency -10%, +10% of their preferred cadence<br>Piano tones (tone frequency: 1319Hz) Inter-onset arrival: 600, 450 and 750ms.	Significant enhancement in gait speed in both Exp and Ct after training and with follow-up.<br>Significant reduction in stride time variability after training, however, effect not seen at follow-up.<br>Significantly shorter inter-step interval with -10% input as compared to +10%, however synchronization variability significantly increased with +10%.<br>No effects on synchronization accuracy.<br>Significantly reduced synchronization variability in hand tapping task with auditory input.<br>Significant enhancement in adaptation index, and phase correction relative to group average was reported.
Chen, et al. [87]	Effects of auditory cueing on walking turns in patients affected from Parkinsonism	6F, 8M (57-67.3)	4	10.6± 5.8 years	Gait velocity, step length, cadence and freezing of gait score	Gait performance with clock and counter clockwise turns, with/without auditory and/or visual cueing and with/without dual task (carrying a tray with cup of water)	Rhythmic auditory cueing at -10% or +10% of preferred cadence	Significant enhancement in gait velocity, freezing of gait score with auditory cueing in both single and dual task conditions.<br>Significant enhancement in gait velocity (dual-task only), step length, cadence and freezing of gait score in audio-visual condition in both single and dual task conditions.

Pau, et al. [119]	Effects of auditory cueing on gait in patients affected from Parkinson’s disease	6F, 20 M (70.4±9)	4	7.5±5.4 years	Gait speed, cadence, stance phase %, swing phase % and double support %, step length, step width, dynamic range of motion for hip flexion/extension, knee flexion/extension, ankle dorsiflexion/plantarflexion, gait variable score (pelvic tilt, pelvic rotation, pelvic obliquity, hip flexion-extension, hip abduction-adduction, hip rotation, knee flexion-extension, ankle dorsi-plantarflexion, foot progression) and gait profile score	Pre-test, gait training for 45 minutes’ session, twice/week for 5 weeks, post-test<br>Home training for 30 minutes’ session, 5 days/ week for 12 weeks, follow up post-test after 17 weeks	Rhythmic auditory cueing (beats) for +10% (if cadence below normality), less than 10% difference (if cadence below but close to normality), at preferred cadence (if cadence above normality)	Significant enhancement in step length, gait speed, cadence, swing phase % after 5 weeks of supervised training and 17 weeks of home training with rhythmic auditory cueing as compared to baseline.<br>Significant enhancement in step width after 17 weeks of training with rhythmic auditory cueing as compared to baseline and 5-week training.<br>Significant reduction in stance phase % (5-week only) and double support % after 5 weeks of supervised training and 17 weeks of home training with rhythmic auditory cueing as compared to baseline.<br>Significant reduction in gait profile score, gait variable score (hip flexion-extension) after 17 weeks of training with rhythmic auditory cueing as compared to baseline and 5-week training. Significant enhancement in gait variable score for (ankle dorsi-plantarflexion) after 17 weeks of training with rhythmic auditory cueing as compared to baseline and 5-week training.<br>Significant enhancement in dynamic range of motion at hip flexion-extension (17-week> 5-week), knee flexion-extension after 5 weeks of supervised training and 17 weeks of home training with rhythmic auditory cueing as compared to baseline.
Zhao, et al. [198]	Effect of rhythmic auditory cueing with google glass on gait in patients affected from Parkinson's disease	3F, 9M (66.8±6.8)	5	13.6± 6.7 years	Cadence, deviation in cadence, stride length, stride length variability, gait speed and freezing of gait (duration/trial)	Gait performance in a wide/narrow 180° turn, full 360° turn, 90° turn track, across a doorway with/without rhythmic metronome cueing at preferred cadence, visual (LED/optic flow)	Rhythmic metronome cueing (80-124 steps/min i.e. preferred cadence)	Significant enhancement in stride length, gait speed (doorway course) with rhythmic metronome cueing.<br>Significant reduction in stride length variability, cadence (narrow and full turn course) with rhythmic metronome cueing.<br>No effect on freezing of gait with rhythmic metronome cueing.<br>Rhythmic metronome cueing preferred as compared to visual cueing by patients.
Baram, et al. [199]	Effects of auditory feedback on gait in patients affected from Parkinson’s disease	2F, 14M (69.9±7.8)	5	6.1±4.6 years	Gait speed, stride length, 10 metres walking test	Pre-test, followed by rhythmic auditory feedback and 15 min follow-up short term residual performance test	Clicking sound generated with gait step	Significant enhancement in gait speed and stride length with rhythmic auditory cueing.<br>Significant enhancement in short-term residual performance with auditory cueing.

Son and Kim [197]	Effect of rhythmic auditory cueing on arm and trunk kinematics during gait in patients affected from Parkinson's disease	8F, 5M (64.8±6.8)	4	64.2± 37.8 months	Arm swing amplitude and trunk rotation	Gait performance with/without rhythmic auditory cueing and/or visual cueing (strips at 40% distance of participant's height)	Rhythmic metronome cueing +20% faster than preferred cadence	Significant enhancement in arm swing amplitude with auditory cueing as compared to visual, audio-visual and no stimuli condition<br>Enhancement in trunk rotation range with audio-visual input as compared to visual, auditory and no stimuli condition.
Song, et al. [120]	Effect of rhythmic auditory cueing on gait and balance in patients affected from Parkinson's disease	Exp: 26F, 30M (65.7±8.1)<br>Ct: 27F, 29M (66.1±7.9)	5	Exp: 6.9± 2.9 years<br>Ct: 6.7± 3.1 years	Stride length, cadence, gait velocity, Unified Parkinson's disease rating scale II, III, 6 minutes walking test and berg balance score	Pre-test, gait training with rhythmic auditory and visual cueing for 30 minutes' session, 5 times/week, for 8 weeks, post-tests at 4 and 8 weeks	Rhythmic auditory cueing (beats) at preferred cadence	Significant enhancements in stride length, gait velocity, six minutes walking distance, berg balance score after 4, 8 weeks of training training with rhythmic auditory cueing and in Exp as compared to Ct.<br>Significant reduction in unified parkinsons disease rating score II and III after 4, 8 weeks of training with rhythmic auditory cueing and in Exp as compared to Ct.
De Icco, et al. [123]	Effect of auditory cueing on gait in patients affected from Parkinson's disease	Exp:<br>Acoustic: 4F, 7M (78.1±6.1)<br>Visual: 6F, 5M (73.2±69)<br>Ct: 12F, 12M (72.1±7.3)	4	Exp:<br>10±3.1 years<br>Visual: 9± 2.4 years<br>Ct: 10.5± 5.2 years	Number of stride, stride duration, stride length, stance % of stride, swing % of stride and gait speed	Pre-test, gait training with/without acoustic, visual stimulus 20 minutes session for 5 sessions/ week for 4 weeks, post-test, 3 months follow up post-test	Rhythmic metronome cueing with frequency between 60-120Hz at preferred cadence	Significant enhancement of gait speed, stride length and reduction in number of strides post acoustic cueing training. However, the effects reduced in the 3 months follow up post-test.
Bukowska , et al. [54]	Effect of auditory cueing on gait and postural stability performance in patients affected from Parkinson's disease	Exp: 15F, 15M (63.4±10.6)<br>Ct: 10F, 15M (63.4±9.6)	4	Exp: 5.5± 3.9 years<br>Ct: 6.7± 4.3 years	Gait velocity, step length, stride length, step width, Stance phase, swing phase, double support %, stride time, cadence, spatial (elongation of step, stride length, increase of velocity, step width), temporal (shortening of stance phase, double support, stride time, increase of cadence,	Pre-test, gait training and postural stability (with eyes closed/open) with auditory cueing for 45 minutes' session, 4 times a week for 4 weeks, therapeutic instrument music performance, patterned sensory enhancement facilitated gait phases, step length, body weight distribution, coordination and reciprocated movements of upper and lower limbs.	Auditory cueing by rhythmic metronome cueing, therapeutic instrument music performance, patterned sensory enhancement Percussion instruments for rhythmic cueing, metronome tone embedded in music	Significant enhancement in swing phase, cadence, step length, gait velocity and stride length after training in Exp as compared to Ct.<br>Significant reduction in postural sway (eyes open, sagittal plane) stance phase, double support, stride time, after training in Exp as compared to Ct.

					extension of swing phase) parameters and Rhomberg's test	Rhythmic auditory cueing enhanced gait speed, step length, walking up and down stairs		
Benoit, et al. [94]	Effect of auditory cueing on gait and motor task performance in patients affected from Parkinson's disease	Exp: 5F, 10M (67.2±7.5)<br>Ct: 10F, 10M (66.4±7.8)	5	Exp: 7.9± 2.7 years	Gait: Cadence, stride length, stride length variability, gait speed, stride time, stride time variability, synchronization accuracy and inter-step interval<br>Hand tapping (BAASTA: Battery for the assessment of auditory sensorimotor timing abilities): Adaptation Index, phase correction, synchronization accuracy and variability	I: Pre-test, 3 training session/ week for 1 month. Three trials, 30 minutes each with 3 phases (10 minutes each) i.e. 1st and 3rd phase cued with music for 8 min, followed by 2 min of no feedback gait, post-test, follow-up 1 month after training<br>II: Hand-tapping BAASTA: Anisochrony detection without tone/music, paced tapping to isochronous sequence/music, synchronization continuation	Music beat cueing adjusted to patient's preferred cadence<br>Patients trained with beat frequency -10%, +10% of their preferred cadence<br>Piano tones (tone frequency: 1319Hz) Inter-onset arrival: 600, 450 and 750ms.	Significant enhancement in gait speed and step length with auditory inputs in Exp, even during the follow-up test.<br>Significant enhancement in synchronization accuracy with isochronous sequences after training.<br>No significant differences in synchronization variability before training and synchronization accuracy and variability with music.<br>Significant enhancement in detection of misaligned beat enhanced after training with follow-up.<br>Exp group had higher thresholds than CT in duration discrimination and improved with training.
Harro, et al. [110]	Effect of rhythmic auditory cueing on gait in patients affected from Parkinson's disease	Exp: 2F, 8M (67.31±1.4)<br>Ct:5F, 5M (46.9±9.4)	8	Exp: 3.7±2.2 years<br>Ct: 4.2±2.4 years	Functional gait assessment, comfortable gait speed, fast gait speed, 6-minute walking distance test	Pre-test, gait training with rhythmic auditory cueing on ground (Exp), speed gait training on treadmill (Ct) for 30 minutes' session/week, for 6 week, 3-month follow up post-test	Rhythmic auditory cueing (+5-10bpm than preferred cadence in following sessions i.e. 105-144bpm)	Significant enhancement in comfortable gait speed, 6-minute walking distance, functional gait assessment after training with rhythmic auditory cueing.<br>Significant enhancement in retention performance for functional gait assessment, comfortable gait speed, fast gait speed, 6-minute walking distance test during 3-month follow up post-test after training with rhythmic auditory cueing.<br>No difference in between Exp and Ct.
Lopez, et al. [88]	Effect of rhythmic auditory cueing on gait in patients affected from Parkinson's disease	3F, 7M (45-65)	6	-	Cadence, stride length, gait speed	Gait performance with/without rhythmic auditory cueing at +25% of preferred cadence	Rhythmic auditory cueing at +25% of preferred cadence (Listenmee®)	Significant enhancement in cadence, stride length, gait speed with rhythmic auditory cueing.
Young, et al. [92]	Effect of rhythmic auditory cueing on gait in	I: Exp: 6F, 4M (64.6±5)	5	3.1± 1.3 years	I: Mean step length, % change stride length, mean step duration, %	I: Gait performance with/without verbal instruction, verbal instruction-metronome cueing, stepping	I: Rhythmic auditory cueing (Ct: 550-649ms, Exp: 600-700ms), foot step feedback on gravel (500, 600, 700ms)	I: Significant reduction in stride length variability, stride duration variability for stepping sound and stepping sound-verbal instructions as compared to

	patients affected from Parkinson's disease	Ct: Healthy<br>6F, 4M<br>(63.9±4)<br>II: same as I<br>III: same as I			change in variability of stride length, duration<br>II: same as I<br>III: same as I	sound, stepping sound-verbal instructions, for small and wide stride length (randomized)<br>II: Gait performance with/without stepping sound, verbal instruction-stepping sound feedback, synthesized gravel sound, synthesized gravel sound-verbal instructions, for small and wide stride length (randomized)<br>III: Gait performance with/without motor imagery, motor imagery-stepping sound feedback, synthesized gravel sound, synthesized gravel sound-motor imagery, for small and wide stride length (randomized)	II: Rhythmic auditory cueing (Ct: 550-649ms, Exp: 600-700ms), foot step feedback on gravel (500, 600, 700ms), synthesized gravel step sound corresponding to plantar force (developed by using ground reaction forces vector to modulate both intensity envelop and central frequency of bandpass filter applied to stochastic noise impulse signal)<br>III: same as II	metronome and metronome-verbal instructions in Exp.<br>Significant reduction in stride length variability for stepping sound and stepping sound-verbal instructions and metronome-verbal instructions in Exp as compared to Ct.<br>No effect of auditory cueing or instructions on mean step duration.<br>II: Significant enhancement in step length with metronome-verbal instruction as compared to synthesized sound, synthesized sound-verbal instructions.<br>No effect of auditory cueing or instructions on mean step duration.<br>Significant reduction in stride length variability with synthesized feedback as compared to footstep feedback-verbal instruction, synthesized feedback-verbal instructions, and Ct group.<br>Significant reduction in stride duration variability in Exp as compared to Ct.<br>III: Significant enhancement in step length (long steps) with stepping sound, stepping sound-verbal instruction as compared to synthesized feedback, synthesized-verbal instructions.<br>Significant enhancement in step length with synthesized feedback in Ct as compared to Exp.<br>No effect of acoustic feedback or instructions on mean step duration.<br>Significant reduction in stride length variability with stepping, synthesized feedback, stepping-verbal instructions.<br>Significant enhancements in stride length with rhythmic auditory cueing (synthesized) and motor imagery together.<br>No effect on stride duration parameters.
Hove, et al. [154]	Effect of auditory cueing on gait performance in patients affected from Parkinson's disease	Exp: 12F, 8M (69.2±7.7)<br>Ct: 2F, 16M (24.7±2.7)	4	Exp: 3.6 years	Step-tone synchronization, Deterended fluctual analysis and self-reported stability on Likert scale (1-7)	Pre-test, gait performance with under counterbalanced: no auditory, fixed rhythmic auditory tempo, interactive rhythmic auditory tempo (Walkmate), post-test for retention	100ms sine-tone from 523-700 Hz<br>Interactive rhythmic cueing directed at period and phase adjustment	Significantly enhanced step-tone synchronization, fractal scaling and self-reported stability in both Exp and Ct groups when interactive auditory input was present as compared to fixed rhythmic auditory input.

Kadivar, et al. [200]	Effect of auditory cueing on gait performance in patients affected from Parkinson's disease	Exp: 3F, 5M (73.2±2.2)<br>Ct: 2F, 6M (70.5±2.2)	5	Exp: 8.9± 1.8 years<br>Ct: 7.5± 1.2 years	Dynamic gait index, unified parkinson's disease rating scale, Tinetti gait and balance tests, time up and go test and freezing of gait questionnaire.	Pre-test, gait training with rhythmic auditory input at 0%, ±10%, ±20% of preferred cadence, for front, side and back steps for 45-60 min, 3 times per week, for 6 weeks, post-test (last day of training, follow up tests 1 week, 4 weeks and 8 weeks)	Rhythmic tone cueing at 0%, ±10%, ±20% of preferred cadence	Significant enhancement in dynamic gait index, Tinetti gait and balance tests and time up and go test with enhancements persisting in post-tests for last day of training, follow up tests 1 week, 4 weeks and 8 weeks.<br>Significant enhancements in unified Parkinson's disease rating scale, freezing of gait questionnaire score in post-tests for last day of training, follow up tests 1 week, 4 weeks.
Rochester, et al. [68]	Effects of rhythmic auditory cueing on gait in patients affected from Parkinson's disease during "on" and "off" medications	19F, 31M (69.2±8.7)	6	8.6± 5.1 years	Gait velocity, stride amplitude, cadence, coefficient of variability for (stride time, double leg support)	Gait performed in "on" and "off" phase of medication cycle (2 weeks apart), with verbal instruction for taking larger steps and with/without rhythmic auditory cueing at preferred cadence	Rhythmic metronome cueing at preferred cadence	Significant enhancement in gait velocity, stride amplitude (no feedback only), cadence during the "off" phase of medication with rhythmic auditory cueing as compared to no feedback and verbal instructions.<br>Significant reduction in coefficient of variability for (stride time, double limb support) during the "off" phase of dopaminergic medication with rhythmic auditory cueing as compared to no feedback.<br>Significant enhancement in gait velocity, stride amplitude (no feedback only), cadence (verbal instruction only) during the "on" phase of medication with rhythmic auditory cueing as compared to no feedback and verbal instructions.<br>Significant reduction in coefficient of variability for (stride time, double limb support) during the "on" phase of medication with rhythmic auditory cueing as compared to no feedback.
Lohnes and Earhart [106]	Effect of auditory cueing and dual-task on gait performance in participants affected from Parkinson's disease	Exp: 7F, 4M (70.2±6.8)<br>Ct: 7F, 4M (70.8±10.4)<br>7F, 4M (24±0.8)	5	Exp: 9±5.3 years	Gait velocity, cadence and stride length	Patients performed gait with/without rhythmic auditory cueing at -10%, +10% of preferred cadence alone or with additional cueing strategy "think about larger strides" with/without -10% and +10% of auditory inputs tone, with/without dual-task "word generation task"	Metronomic cueing at -10% or +10% of preferred cadence.	Significantly enhanced gait velocity and stride length in Exp within combined condition of additional cues and auditory inputs.<br>Significant increase in stride length in the dual-task setting wit auditory input and additional cues.<br>Modulated auditory input affected gait parameters of Ct.
Ford, et al. [141]	Effects of auditory cueing on gait and treadmill	Exp I: 10M (67.1±4)<br>Exp II: 10M (67.9±6.3)	7	Exp I: 3.7± 4.1 years	Step length, stride length, cadence, 6-meter walk time, distance, gait speed	Participants trained in gait on a treadmill with (Exp I)/without (Ct) rhythmic music cueing for 3 days/ week	Rhythmic music cueing	Significant enhancement in step length, stride length, 6 metre walk time and time up and go test (8th week only) for both 4th and 8th week post-tests after training with auditory cueing and treadmill training.

	training in patients affected from Parkinson’s disease	Ct: 10M (68.6±5.2)		Exp II: 4.4± 2.3 years<br>Ct: 7.4± 3.4 years	and time up and go test	and home training 3 days/week for 4 weeks, followed by 4 weeks of self-training Exp II. Ct group trained for walking 6 days/ week for 4 weeks.		Enhancement in gait speed, 6-minute walk distance, cadence (8th week only) for both 4th and 8th week post-tests after training with auditory cueing and treadmill training.
Espay, et al. [89]	Effects of auditory cueing on gait in patients affected from Parkinson’s disease	5F, 9M (50-79)	5	-	Gait velocity, cadence and stride length	Gait training for 30 minutes a session (evaluation and training in each session i.e. total 24 sessions), 3 sessions/ week for 8 weeks, gait training by auditory cueing tempo increased in middle of training by +10bpm	Rhythmic auditory cueing (5 parts: melody, chords, bass, percussion) music superimposed by metronome +5 beat increments from 60-165bpm	Significant enhancement in gait velocity, cadence and stride length after training with rhythmic auditory cueing.
Lim, et al. [11]	Effect of rhythmic auditory and visual cueing in gait for patients affected from Parkinson’s disease	9F, 6M (73.3±11.7)	4	12.1± 4.2 years	Gait velocity, stride length, cadence	Pre-test, home gait training for 30 minutes’ session/day for (at least) 2 weeks with virtual reality device, testing with/without device, visual, audio-visual cueing, post-test after 2 weeks training	Rhythmic auditory cueing for stepping sound at preferred cadence	Significant enhancement in gait velocity and stride length with combined audio-visual cueing.<br>Significant enhancement in immediate retention measurement without device for gait velocity and stride length.
Arias and Cudeiro [29]	Effects of rhythmic auditory cueing on gait and physical activity for patients affected from Parkinson’s disease	Early: 28F, 48M (67.5)<br>Late: 37F, 40M (69)	7	Early: 4-11 years<br>Late: 4-12 years	Percentage of time on static, dynamic activity, sitting, standing, gait, walking periods (>5, >10 seconds/hour)	Pre-test, gait training at home with rhythmic audio-visual cueing for 9 sessions of 30 minutes each over 3 weeks, under the supervision of therapist, post-test follow up at 9 weeks (early), 6 weeks (late)	Rhythmic auditory cueing (beep)	Significant enhancement in dynamic, static activities, gait and walking periods (>5, >10 seconds/hour) with rhythmic auditory cueing.<br>Patients preferred rhythmic auditory cueing as the medium for cueing as compared to visual cueing modality.
Chaiwanichsiri, et al. [82]	Effects of auditory cueing on gait in patients affected from parkinso Parkinson’s disease nism	Exp: Freezing of gait: 4F, 6M (68.2±8)<br>Exp: No freezing: 3F, 6M (64.4±9.5)<br>Ct: 2F, 8M (70.2±6.8)	6	-	Gait velocity, cadence, step length, turnaround time and freezing episodes	Patients performed gait at preferred cadence, followed by trials at +10% cadence with/without auditory cueing	Tone with wave frequency 4.625 Hz, deliver at pulses of 50ms and inter-pulse duration customized to obtain desired stimulation frequency	Significantly enhanced gait velocity, stride length and cadence in presence of auditory input +10% as compared baseline auditory feedback at preferred cadence.<br>Significantly reduced episodes of freezing in presence of auditory cueing.<br>Significantly reduced turnaround time in all groups in presence of auditory input.

de Bruin, et al. [95]	Effects of auditory cueing on gait in patients affected from Parkinson's disease	Exp: 5F, 6M (64.1±8.1) Ct: 6F, 5M (67±8.1)	8	Exp: 6.4± 4.2 years Ct: 4.5± 3.3 years	Gait velocity, cadence, stride time, stride length and Unified Parkinson's diseases rating scale	Pre-test, gait training 30 min session, 3times/week for 13 weeks, post-test With/without auditory cueing, dual-task (arithmetic task)	Music with tempo to cadence matched characteristics	Significant enhancements in gait velocity, stride time, cadence and motor symptoms with auditory cueing in Exp as compared to Ct. Enhancement in gait velocity and cadence with dual task with auditory cueing.
Elston, et al. [96]	Effects of auditory cueing on gait and quality of life in patients affected from Parkinson's disease	Early intervention: 8F, 13M (71.5±11.3) Late intervention: 5F, 15M (70.4±8.7)	8	-	Gait speed, Parkinson's disease questionnaire, fall assessment and Short form 34 version questionnaires	Pre-test, patients in early intervention acquainted to metronome for 5-10 min, tests at 4, 10, 14 weeks, Post-test Late intervention group introduced to metronome at week 10	Metronome cueing with beat frequency adjusted to preferred cadence	Enhancement in gait speed in early intervention group as compared to late intervention. No differences in outcomes from parkinson's disease questionnaire, fall assessment and Short form 34 version questionnaires
Rochester, et al. [85]	Effects of auditory cueing on gait in patients affected from Parkinson's disease	Early intervention: 28F, 48M (61.5-72) Late intervention: 37F, 40M (62.5-73)	8	Early: 7 (4-11) years Late: 8 (4-12) years	Gait speed, step length and cadence	Visual, auditory and somatosensory input (randomized) with/without dual task (carrying a tray) Early intervention: 30min for 9 sessions over 3 weeks, the next 3 weeks no training was given. Late intervention: No training for first 3 weeks, 30min for 9 sessions over 3 last weeks 6-week follow-up for both groups	Rhythmic beep cueing at preferred cadence	Significant enhancement in step length and gait speed in dual/ single task condition with training by auditory input. Enhancement of cadence with training by auditory input. Retention evident in 6 weeks follow up for the gait parameters.
Picelli, et al. [102]	Effects of auditory cueing on gait in patients affected from Parkinson's disease	3F, 5M (65.1±4.7)	4	6.5± 1.5 years	Stride length, stride time, cadence, gait speed, single support duration, double support duration, single/double support duration, coefficient of variation of stride time, hip, knee, ankle sagittal plane range of motion and maximal values within pull, push-off phase of hip	Gait performed with/without auditory cueing at -10%, 0%, +10% of preferred cadence	Rhythmic metronome cueing at -10%, 0%, +10% of preferred cadence	Significant enhancement on stride length, stride time, cadence, gait speed, double support duration, single/double support duration and coefficient of variation of stride time. With highest effect of +10% auditory cueing. Significant reduction in ankle sagittal plane range of motion, and enhancement in pull-off phase hip joint power.

Rochester, et al. [122]	Effects of auditory cueing on gait in patients affected from Parkinson's disease	3F, 18M (76.4±12.9)	6	-	Gait speed, step amplitude, step frequency, tandem stance and unified parkinsonian disease rating scale II and III	Pre-test, 30 min intervention for 9 sessions for 3 weeks with auditory cueing and with/without dual task (carrying a tray with glass of water), post-test<br>With instructions "take a big step in beat time"	Rhythmic metronome cueing at preferred cadence	Significant enhancement in gait speed, for both single/ dual task condition with auditory input.<br>Significant enhancement in step frequency in single task condition with auditory input.<br>Significant reduction in step amplitude in both single and dual task condition with auditory input.<br>Enhancement in step frequency with dual task and auditory input.<br>Significant enhancement in unified parkinsonian disease rating scale II and III.<br>Enhancement in tandem stance after treatment intervention.
Frazzitta, et al. [103]	Effects of auditory cueing with treadmill training on gait in patients affected from Parkinson's disease	Exp: I: 12F, 8M (71±8)<br>II: 11F, 9M (71±7)	6	Exp I: 13.2± 4.1 years<br>Exp II: 12.9±4.6 years	6-minute walking test distance, gait speed, Unified parkinson's disease rating scale, stride cycle and freezing of gait questionnaire	Pre-test, gait training on treadmill for 20 minutes/day for 4 weeks (28 sessions) with (Exp I)/without treadmill (Exp II) and with visual and auditory cueing, post-test	Rhythmic auditory cueing (synchronized with visual cueing)	Significant enhancement in 6-minute walking test distance, gait speed and stride cycle in Exp I as compared to Exp 2.<br>Significant reduction in freezing of gait score in Exp I as compared to Exp II.<br>No effect on unified parkinsons disease rating scale on both Exp I and Exp II.
Rochester, et al. [81]	Effects of cueing k on gait in patients affected from Parkinson's disease	9M (74.8±6.4)	4	6.1± 6.1 years	Gait speed, stride amplitude, cadence, coefficient of variation of step time, double limb support time	Pre-test, gait performance with/without auditory input, with/without dual task (carrying a tray with glass of water)<br>With different instructions "step in beat time", "take big step in beat time"	Rhythmic beep cueing at preferred cadence	Significant enhancement in gait speed, stride amplitude with walking instructions "big step in beat time" and auditory input in both single and dual task conditions. The enhancements were higher for single task as compared to dual task setting.<br>Reduced coefficient of variation of step time and coefficient of variation of double limb support time with auditory input, single/dual task, and additional instructions. Higher reduction for "big steps in beat time".<br>Enhanced cadence for auditory input in both single and dual task conditions.
Bryant, et al. [108]	Effects of auditory cueing on gait in patients affected from Parkinson's disease	4F, 17M (72±10.3)	4	6.6±4.3 years	Gait speed, cadence, stride length, double support time and base of support	Gait performed with rhythmic auditory cueing at 0%, +25% of preferred cadence, followed by 1 week of self-home training, 30 minutes per day, post-test	Rhythmic auditory cueing at 0% and +25% of preferred cadence	Significant enhancement in gait speed, stride length with rhythmic auditory cueing.<br>Significant retention in gait speed, stride length, double support time, 1 week follow up test with auditory cueing training.<br>Enhancement in cadence with auditory cueing both during initial testing and post 1-week training with auditory cueing.
Ma, et al. [97]	Effects of auditory cueing on rhythmic	11F, 9M (66.4±6.2)		3.7± 2.5 years	Movement time, amplitude of peak velocity, deceleration	Participants performed reaching task with/without auditory input (marching,	Rhythmic marching auditory input (96-100 bpm), volume (62.4±3.2 decibels) and random	No effect on movement variables with marching auditory input as compared to no attention, no sound conditions.

	reach movements in patients affected from Parkinson's disease				time and number of movement units	weather forecast sound), and with/without paying attention to the sound	weather forecast auditory input, volume (67.4±4.2 decibels)	Significantly poorer performance in weather forecast condition on arm movement variables with as compared to no attention, no sound conditions.
Nieuwboer, et al. [28]	Effects of auditory cueing on turn speed in patients affected from Parkinson's disease	Freezers: 29F, 39M (67.3±6.9) Non-freezers: 26F, 39M (66±8.1)	7	Freezers: 8.7± 4.7 years Non-freezers: 7.8± 5.1 years	Mean turning time	Pre-test, functional gait performed, participants picked up a tray with a cup of water and turned 180º while walking, with auditory, visual, somatosensory input (randomized), post-test	Rhythmic auditory cueing at preferred cadence	Significantly enhanced turning time with auditory input as compared to visual input, but no difference with somatosensory input.<br>Short term carry-over evident after the treatment duration in post-test (with all three inputs trained).
Arias and Cudeiro [101]	Effects of auditory cueing on gait in patients affected from Parkinson's disease	Exp: 10F, 15M (65.9±7.6)- 9 patients' severe (71.3±3.2), 16 patients' mild (62.8±7.8) Ct: 6F, 5M (65.7±7.6)	5	Severe: 12.8± 7 years Mild: 6.7±4.6 years	Cadence, gait velocity, step amplitude, coefficient of variation for step amplitude and stride time	Patients performed gait with/without sensory rhythmic input from auditory, visual and audio-visual condition, with frequency ranging from 70-110% increment/decrement at ±10%	Rhythmic tone with wave frequency of 4625 Hz delivered at frequency ranging from 70-110% increment/decrement at ±10%	Significantly enhanced cadence, step amplitude in severely affected Parkinson's patients with auditory and audio-visual cueing.<br>Significantly enhanced gait cadence, velocity, stride length with increased auditory input i.e. 70%, 80%, 90%...<br>Significantly reduced coefficient of variation for stride time in severely affected Parkinson's patients with auditory and audio-visual cueing.<br>Significant enhancement in cadence, step amplitude in Ct with auditory cueing.<br>Reduced coefficient of variation of step amplitude and enhanced gait velocity in severely affected Parkinson's patients with auditory and audio-visual cueing.
Baker, et al. [114]	Effects of auditory cueing on gait in patients affected from Parkinson's disease	Exp: 9F, 5M (69.2±3.3) Ct: 7F, 5M (71.5±2.5)	7	Exp: 6.6± 3.2 years	Gait speed, coefficient of velocity for (step time, double limb support time)	Pre-test, functional gait performance with/without auditory cueing -10% of preferred cadence, attentional cue instructions "try to take big steps", together "take a big step with the beat", and with/without a dual task (a tray with 2 cups of water on top), post-test	Rhythmic auditory cueing at -10% of preferred cadence	Significant enhancement in gait speed for Exp with rhythmic auditory cueing and verbal instructions together under both single and dual task conditions.<br>Significant reduction in coefficient of variability [step time, double limb support (single task only)] for Exp with rhythmic auditory cueing and verbal instructions together under both single and dual task conditions.<br>Significant enhancement in gait speed for Ct with rhythmic auditory cueing and verbal instructions together under single task condition.
Rochester, et al. [118]	Effects of auditory cueing on gait in patients affected from	65F, 88M (67±7.5)	8	8.2±5 years	Gait velocity, step amplitude and step frequency	Pre-test, functional gait performed with/without auditory, visual or somatosensory cueing (randomized), with/without	Rhythmic auditory cueing at preferred cadence	Significant enhancement in step amplitude (dual task only), gait velocity and step frequency with auditory cueing and under both single and dual task conditions, as compared to no auditory cueing.

	Parkinson's disease					dual task (tray with two cups of water), Post-test, 3-weeks post-test		No effects evitable in 3-weeks post-test retention measurement.
Baker, et al. [113]	Effects of auditory cueing on gait in patients affected from Parkinson's disease	Exp: 9F, 6M (68.8±3.3)<br>Ct: 7F, 4M (71.5±2.5)		6.5±3.2 years	Gait speed, step amplitude and step frequency	Pre-test, functional gait performance with/without auditory cueing -10% of preferred cadence, attentional cue instructions "try to take big steps", together "take a big step with the beat", and with/without a dual task (a tray with 2 cups of water on top), post-test	Rhythmic auditory cueing at -10% of preferred cadence	Significant effect of auditory cueing and attentional cue "big steps with beat" on step frequency in gait speed, step amplitude, step frequency (dual task only) in Exp in both single and dual task conditions.<br>Significant effect of auditory cueing and attentional cue "big steps with beat" on step frequency in gait speed (single task only), step amplitude, step frequency in Ct in both single and dual task conditions.<br>Non-significant effects on gait speed, step amplitude and step frequency with auditory cueing only.<br>Effects not evitable once the auditory cueing was removed, in post-test.
Hausdorff, et al. [111]	Effects of auditory cueing on gait performance in patients affected from Parkinson's disease	Exp: 13F, 16M (67.2±9.1)<br>Ct: 14F, 12M (64.6±6.8)	5	-	Stride time, gait speed, stride length, swing time, stride time variability and swing time variability	Pre-test, gait performance with/without rhythmic auditory cueing at preferred cadence, +10%, Post-test 2 and 15 min short term retention test	Rhythmic auditory cueing at 0% and +10% of preferred cadence	Significant enhancement in gait speed, stride length and swing time with auditory input in Exp.<br>Significant enhancement in Stride time, gait speed, stride length, swing time at +10% input. Significant reduction in stride time variability and swing time variability in Exp at +10% input.<br>Significant enhancement in Stride time, gait speed, stride length, swing time for immediate retention measurements for Exp for auditory cueing and at +10% input.
Nieuwboer, et al. [86]	Effects of auditory cueing on gait and posture in patients affected from Parkinson's disease	Early intervention: 28F, 48M (61.5-72)<br>Late intervention: 37F, 40M (62.5-73)	8	Early: 7 (4-11)<br>Late: 8 (4-12)	Posture and gait score, walking speed, step length, step frequency, functional reach, single stance, tandem stance, time-up and go test, freezing of gait questionnaire, Nottingham extended activities of daily life scale, falls efficacy scale, Parkinson's disease questionnaire, carer stain index and number of fall recording	Similar therapy in early and late intervention groups<br>Early intervention: 30min for 9 sessions over 3 weeks, the next 3 weeks no training was given.<br>Late intervention: No training for first 3 weeks, 30min for 9 sessions over 3 last weeks<br>6-week follow-up for both groups<br>With auditory, visual, somatosensory input (randomized) at preferred cadence<br>Tests at 1, 3, 6 and 12 weeks	Rhythmic auditory cueing at preferred cadence	Significant enhancement in posture and gait score for early and late intervention group.<br>Significant reduction in severity of freezing.<br>Significant enhancement in gait speed, step length and times balance tests for both groups.<br>Significantly enhanced confidence for carrying out functional activities post training.<br>Patients had higher compliance with auditory cueing (67%).

Willems, et al. [84]	Effects of rhythmic auditory cueing on turning in patients affected from Parkinson's disease	Exp: Freezers: 9 (62.6±3.9) Non-freezers: 10 (6.6±6.2) Ct: 9 (68.1±7.3)	5	Non-freezers: 6.2± 3 years Freezers: 11.8± 5.7 years	Steps (number, time, height, width, length), step length, step width, step duration, coefficient of variation of step duration	Gait performance while turning with/without rhythmic auditory cueing	Rhythmic metronome cueing at preferred cadence	Significant reduction in coefficient of variation of step duration for both Exp I and II with auditory cueing.<br>No effects on step length, width and duration.
Chester, et al. [107]	Effects of auditory cueing on gait performance in patients affected from Parkinson's disease	10F, 19M (67.8±10.9)	5	-	Gait speed, relative gait speed stride time, stride length and single, double limb support	Gait performed with rhythmic auditory cueing at -10% and +10% of preferred cadence (randomized).	Rhythmic auditory cueing at ±10% of auditory cueing.	Significant enhancement of gait speed, relative gait speed, stride length, and single limb support with +10% of auditory cueing. Reduction in double limb support and stride time.<br>Significant reduction of gait speed, relative gait speed and enhancement in stride time for -10% of auditory cueing.
Willems, et al. [83]	Effects of auditory cueing on gait performance in patients affected from Parkinson's disease	Exp: Non-freezers: 10 (60.6±6.2) Freezers: 10 (68.4±6.9) Ct: 10 (67.2±9.1)	4	Non-freezers: 6.2±3 years Freezers: 11.8±5.7 years	Step frequency, gait speed, stride length and double support %	Pre-test, gait performance at 0%, -20%, -10%, +10%, +20% of rhythmic auditory input (randomized), post-test	Rhythmic metronome cueing at preferred cadence i.e. 0%, -20%, -10%, +10%, +20%	Significant enhancement in gait speed, step frequency, stride length in Exp with auditory input at 0%, step frequency significantly enhanced in +10%, +20% and significantly reduced in -10%. Similarly, for the Ct in both step frequency and gait speed at 0%, -10%, +10%, +20%.<br>Freezers and non-freezers showed similar response to rhythmic auditory inputs.
del Olmo, et al. [99]	Effects of auditory cueing on gait and finger tapping in patients affected from Parkinson's disease	Exp: 4F, 5M (61.2±5.5) Ct: 3F, 2M (63.2. ±4.8)	4	Exp: 5.7± 1.9 years	Gait velocity, cadence, step length, coefficient of variability of 2 consecutive steps, tapping frequency, coefficient of variation of interval of 2 consecutive taps, PET scan	Pre-test, gait training with rhythmic auditory cueing, rehabilitation for 1 hour/day, 5 days/ week for 4 weeks	Metronome cueing at rates between 30 and 150 bpm for gait Metronome at rates between 0.5 and 4 Hz for finger tapping.	Significantly reduced coefficient of variability for steps and finger tapping with auditory input as compared to Ct.<br>No effect on gait velocity, cadence and step length.<br>Significant hypo metabolism for Exp in right parietal and temporal lobes, left temporal and frontal lobes.<br>Significant hypermetabolism in Exp in left cerebellum.<br>Significant metabolic increment in Exp in right cerebellum, right parietal and temporal lobes.

Jiang and Norman [98]	Effects of auditory cueing on gait-initiation in patients affected from Parkinson's disease	Freeze history: 5F, 2M (67±13)<br>No-freeze history: 7M (70±7)	5	Freeze: 6.1± 5.4 years<br>No-freeze: 3.4± 1.4 years	Measures of magnitude: Posterior horizontal force, length of 1st and 2nd step, gait velocity and push-off force during gait initiation	Gait initiation and performance for 30 metres	High pitched beep at 40ms duration, interval set in auditory inputs per preferred gait.	No effect of auditory inputs on measures of magnitude, push-off force and gait velocity.<br>No effects of auditory inputs on key events timing in gait initiation<br><br>Significant enhancement in coefficient of variability with auditory cueing in between pre-posttests.<br>No difference in coefficient of variability in Exp and Ct group.<br>Significant enhancement in gait velocity, step length and cadence with auditory cueing.
del Olmo and Cudeiro [109]	Effects of auditory cueing on gait in patients affected from Parkinson's disease	Exp: 7F, 8M (61.7±5.2)<br>Ct: 4F, 11M (63.1±4.2)		Exp: 7.2± 4.3 years	Gait velocity, step length, cadence, coefficient of variability i.e. temporal variability of gait	Gait performed at preferred and fast speed with and without a dual-motor task (thumb apposition task) for 1 hour/day for 5 days/week for 4 weeks while reproducing heard auditory cueing or while receiving auditory cueing	Metronome cueing: 60, 90, 120, 150 bpm during reproduction task and synchronized task.	Significant enhancement in coefficient of variability with auditory cueing in between pre-posttests.<br><br>No difference in coefficient of variability in Exp and Ct group.<br><br>Decrease in gait velocity, step length and cadence with auditory cueing.
Rochester, et al. [116]	Effects of auditory cueing on gait in patients affected from Parkinson's disease	Exp: 8F, 12M (64.6±7.9)<br>Ct: 4F, 6M (63.5±7)	6	Exp: 10± 1.6 years	Step length, step frequency, walking speed, time duration and cadence	Complex functional walking and sitting task under single and dual-motor task (carrying a tray) condition	Rhythmic auditory cueing generated according to preferred speed of patients.	Significant enhancement in step length of dual-motor task with auditory cueing as compared to Ct group.<br><br>Enhancement in walking speed for patients in dual-motor task with auditory cueing.<br><br>No difference in step length and walking speed in single task conditions.<br><br>No difference in step frequency, time duration and cadence in both single and dual-motor conditions.
Suteerawattananon, et al. [112]	Effects of auditory cueing on gait in patients affected from Parkinson's disease	10F, 14M (68.9±10.4)	5	6.9±4.4 years	Gait speed, cadence and stride length	Gait performed with/without visual and/or auditory cueing	Rhythmic metronome cueing +25% of preferred cadence	Significant enhancement of gait speed and cadence with auditory input.<br><br>No effect on stride length with auditory input
Cubo, et al. [201]	Effects of auditory cueing on gait in	4F, 8M (65.8±11.2)	8	12.4± 7.3 years	Total freezing instances, time, average duration of a	Pre-test, gait performance with rhythmic auditory cueing at preferred cadence (post-test	Rhythmic metronome cueing at preferred cadence	Significant reduction in walking time during post-test 2 as compared to post-test 1 with rhythmic auditory cueing

	patients affected from Parkinson's disease				freeze, gait time, total procedure time	1) and home training daily for 1 week, post-test 2		Reduction in total procedure time, average duration of freeze during post-test 2 as compared to post-test 1 with rhythmic auditory cueing.<br><br>Significant enhancement in walking time during post-test 1 with rhythmic auditory cueing<br>Reduction in number of freeze instances during post-test 1 with rhythmic auditory cueing.
Howe, et al. [90]	Effects of auditory cueing on gait in patients affected from Parkinson's disease	2F, 9M (30-67)	6	-	Cadence, gait velocity and stride length	Patients performed gait with auditory input and at 85%, 92.5%, 100%, 107.5%, 115% of mean preferred cadence	Music motor cueing adjusted for speed by time interval adjusted between consecutive heel strikes	Significant enhancement in gait velocity, stride length, heel on-toe-off distance.<br><br>Significantly reduced symmetry deviation.<br><br>Enhanced cadence with auditory cueing.
Freedland, et al. [202]	Effects of auditory cueing on gait in patients affected from Parkinson's disease	5F, 11M (74±7.2)	4	-	Gait cycle time, double support, step length, base of support, cadence, step-extremity ratio, Functional ambulation performance score, mean normalized velocity	Pre-test, gait performed with rhythmic auditory input at 0% and +10% of preferred cadence, post-test	Rhythmic metronome cueing at 0% and +10% of preferred cadence	Significant enhancement in step length, and step extremity ratio and reduction in gait cycle time, double support with auditory cueing.
McIntosh, et al. [104]	Effects of auditory cueing on gait in patients affected from Parkinson's disease	With meds: 6F, 15 M (71±4)<br>24h post meds: 4F, 6M (73±3)<br>Ct: 6F, 4M (72±5)	4	Exp: 7.5 years	Gait velocity, stride length, cadence and cadence-auditory stimulus synchronization	Gait performance by participants with pre-test, with and without normalized auditory and at +10% of preferred cadence, post-test.	0%, +10% of basic tempo for metronome adjusted at patients preferred cadence.	Significant enhancement in gait velocity, cadence and stride length with +10% auditory stimulus.<br><br>Significantly enhanced synchronization in Ct, but synchronization not evident in both Exp groups.
Thaut, et al. [105]	Effects of auditory cueing on gait in patients affected from Parkinson's disease	Exp: 5F, 10M (69±8)<br>Ct: Self-paced: 3F, 11M (74±3)<br>No training: 3F, 11M (71±8)	5	Exp: 7.2±4 years<br>Ct: 5.4±3 years<br>No training: 8.5±4 years	Gait velocity, stride length, cadence and Electromyogram amplitude variability (Gastrocnemius, tibialis anterior, vastus medialis)	Pre-test/ training for 30 min/day for 3 weeks/ post-tests<br>Walking with rhythmic auditory cueing on flat surface, incline stair steps	Rhythmic auditory cueing embedded in music beat structure for: preferred cadence, quick (normal +5-10%), fast (quick +5-10%) pace	Significant enhancement in gait velocity, stride length and cadence in Exp.<br><br>Re-production of performance parameters evident after training in absence of auditory stimuli.<br><br>Significant reduction in electromyogram amplitude variability of tibialis anterior and vastus medialis muscle.

Exp: experimental group, Ct: Control group

Table 2 Individual Pedro scores

Study	PEDRO Score	Point estimates & variability	Between group comparison	Intention to treat	Adequate follow-up	Blind assessors	Blind therapists	Blind subjects	Baseline comparability	Concealed allocation	Random allocation	Eligibility criteria
Dotov et al. (2017)	6	1	1	0	1	0	0	0	1	1	1	1
Dalla Bella et al. (2017)	6	1	1	0	1	0	0	0	1	1	1	1
Pau et al. (2016)	4	1	1	0	1	0	0	0	0	0	1	1
P.-H. Chen et al. (2016)	4	1	1	0	1	0	0	0	0	0	1	1
Baram et al. (2016)	4	1	1	0	1	0	0	0	0	0	1	1
Zhao et al. (2016)	5	1	1	0	1	0	0	0	1	0	1	1
Bukowska et al. (2015)	4	1	1	0	1	0	0	0	0	0	1	1
De Icco et al. (2015)	6	1	1	0	1	0	0	0	1	1	1	1
J. Song et al. (2015)	5	1	1	0	1	0	0	0	1	0	1	1
Son and Kim (2015)	4	1	1	0	1	0	0	0	0	0	1	1
Benoit et al. (2014)	6	1	1	0	1	0	0	0	1	1	1	1
Harro et al. (2014)	8	1	1	0	1	1	1	0	1	1	1	1
Lopez et al. (2014)	6	1	1	0	1	0	0	0	1	1	1	1
Young et al. (2014)	5	1	1	0	1	0	0	0	1	0	1	1
Hove et al. (2012)	4	1	1	0	1	0	0	0	0	0	1	1
Kadivar et al. (2011)	5	1	1	0	1	0	0	0	1	0	1	1
Lohnes and Earhart (2011)	5	1	1	0	1	0	0	0	1	0	1	1
Rochester et al. (2011)	6	1	1	0	1	0	0	0	1	1	1	1
Chaiwanichsiri et al. (2011)	7	1	1	0	1	1	0	0	1	1	1	1
Arias and Cudeiro (2010)	5	1	1	0	1	0	0	0	1	0	1	1
de Bruin et al. (2010)	8	1	1	0	1	1	1	0	1	1	1	1
Elston et al. (2010)	8	1	1	0	1	1	1	0	1	1	1	1
Espay et al. (2010)	4	1	1	0	1	0	0	0	0	0	1	1
Ford et al. (2010)	5	1	1	0	1	0	0	0	1	0	1	1
Picelli et al. (2010)	4	1	1	0	1	0	0	0	0	0	1	1
Rochester, Baker, et al. (2010)	8	1	1	0	1	1	1	0	1	1	1	1
Rochester, et al. (2010)	6	1	1	0	1	0	0	0	1	1	1	1
Bryant et al. (2009)	4	1	1	0	1	0	0	0	0	0	1	1
Rochester et al. (2009)	4	1	1	0	1	0	0	0	0	0	1	1
Nieuwboer et al. (2009)	7	1	1	0	1	1	0	0	1	1	1	1
Frazzitta et al. (2009)	6	1	1	0	1	0	0	0	1	1	1	1
Arias and Cudeiro (2008)	4	1	1	0	1	0	0	0	0	0	1	1
Baker et al. (2008)	6	1	1	0	1	0	0	0	1	1	1	1
Nieuwboer et al. (2007)	8	1	1	0	1	1	1	0	1	1	1	1
Baker et al. (2007)	6	1	1	0	1	0	0	0	1	1	1	1
A.-M. Willems et al. (2007)	5	1	1	0	1	0	0	0	1	0	1	1
Hausdorff et al. (2007)	5	1	1	0	1	0	0	0	1	0	1	1
Rochester et al. (2007)	8	1	1	0	1	1	1	0	1	1	1	1
Y. Jiang and Norman (2006)	5	1	1	0	1	0	0	0	1	0	1	1

Chester et al. (2006)	5	1	1	0	1	0	0	0	1	0	1	1
del Olmo et al. (2006)	4	1	1	0	1	0	0	0	0	0	1	1
A.-M. Willems et al. (2006)	4	1	1	0	1	0	0	0	0	0	1	1
del Olmo and Cudeiro (2005)	4	1	1	0	1	0	0	0	0	0	1	1
Rochester et al. (2005)	6	1	1	0	1	0	0	0	1	1	1	1
Cubo et al. (2004)	8	1	1	0	1	1	1	0	1	1	1	1
Suteerawattananon et al. (2004)	5	1	1	0	1	0	0	0	1	0	1	1
Howe et al. (2003)	6	1	1	0	1	0	0	0	1	1	1	1
Freedland et al. (2002)	4	1	1	0	1	0	0	0	0	0	1	1
McIntosh et al. (1997)	4	1	1	0	1	0	0	0	0	0	1	1
M. H. Thaut et al. (1996)	5	1	1	0	1	0	0	0	1	0	1	1

1: point awarded, 0: no points awarded

Meta-analysis Figures

on individual
ge's g (boxes) and 95%
d) represents pooled
positive effect size
rs, NFz: Non-Freezers,
: Fast paced training, I:
ep frequency: number

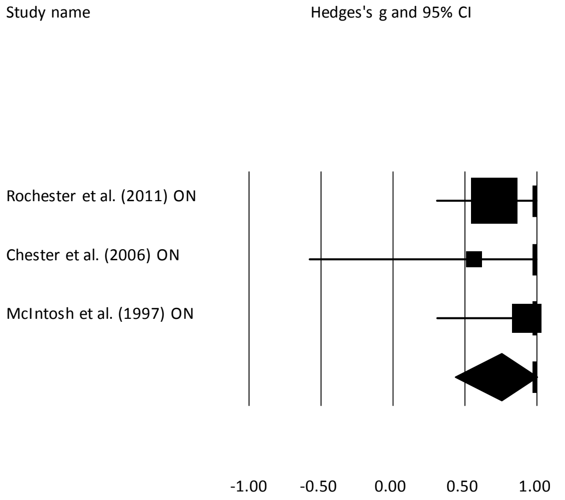

Figure 1 Gait velocity evaluated in ON medication condition

Favours A Favours B

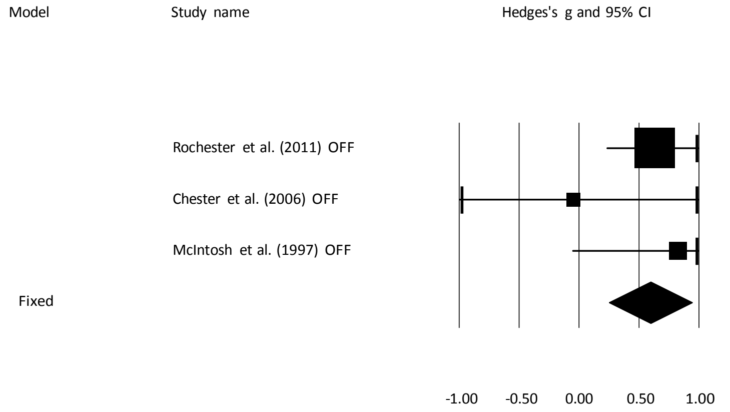

Figure 2 Gait velocity evaluated in OFF medication condition with rhythmic auditory cueing

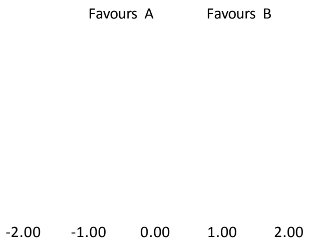

Figure 3 Gait velocity evaluated on treadmill with rhythmic auditory cueing

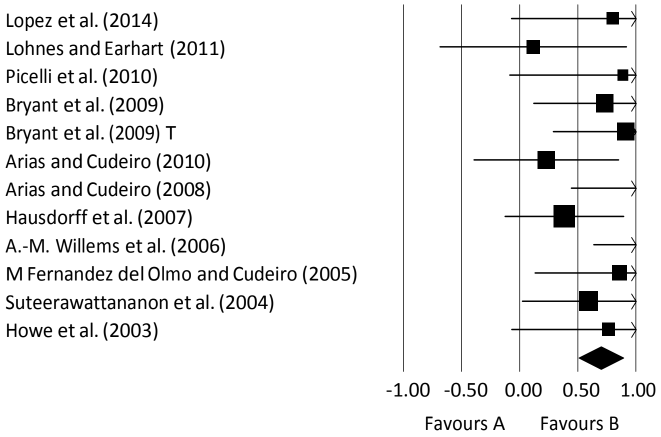

Figure 4 Gait velocity evaluated with fast paced rhythmic auditory cueing (pace of stimuli determined with reference to patient's preferred cadence)

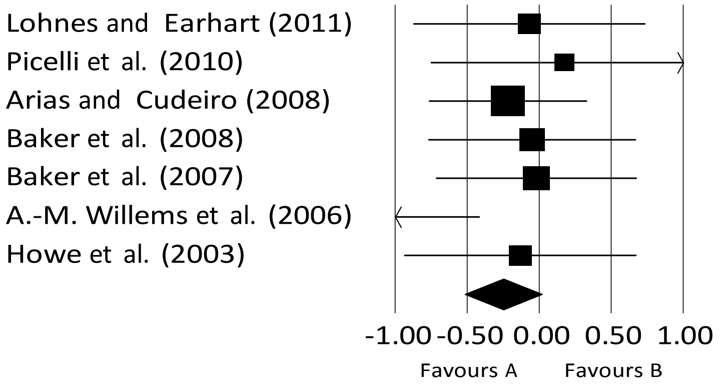

Figure 5 Gait velocity evaluated with slow paced rhythmic auditory cueing (pace of stimuli determined with reference to patient's preferred cadence)

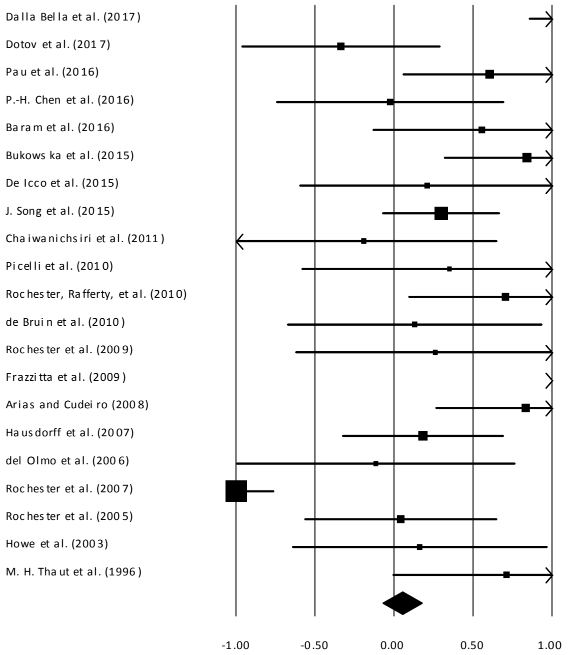

Figure 6 Gait velocity evaluated with un-modulated rhythmic auditory cueing

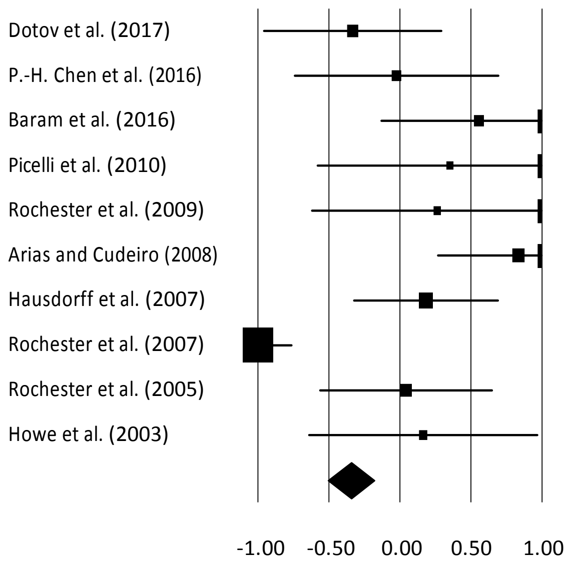

Figure 7 Gait velocity analysed with un-modulated rhythmic auditory cueing without training

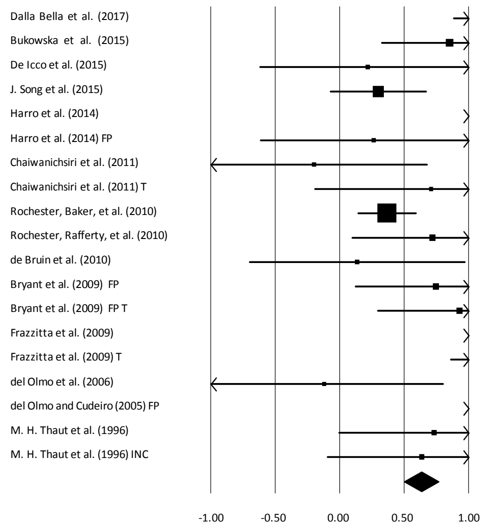

Figure 8 Gait velocity analysed with rhythmic auditory cueing with training

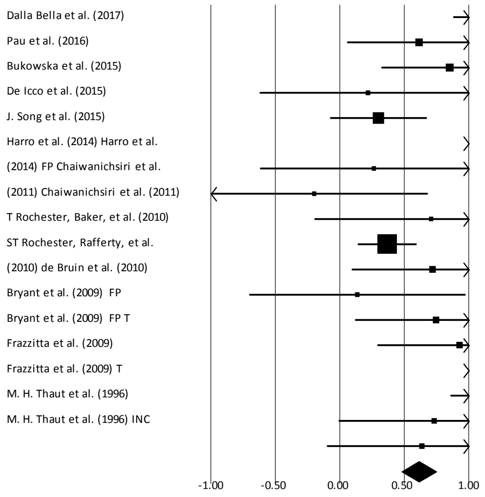

Figure 9 Gait velocity analysed with training for more than 45 minutes with rhythmic auditory cueing

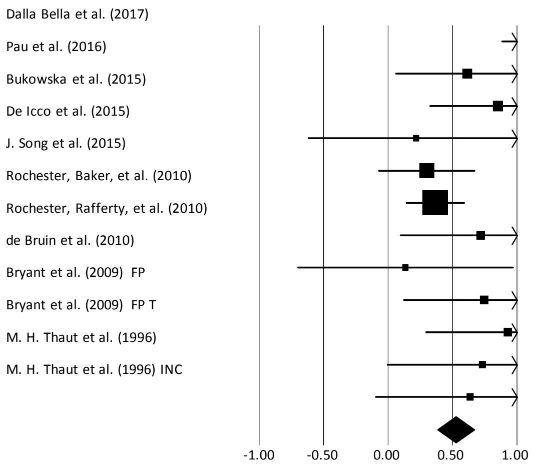

Figure 10 Gait velocity analysed with training for 30-45 minutes with rhythmic auditory cueing

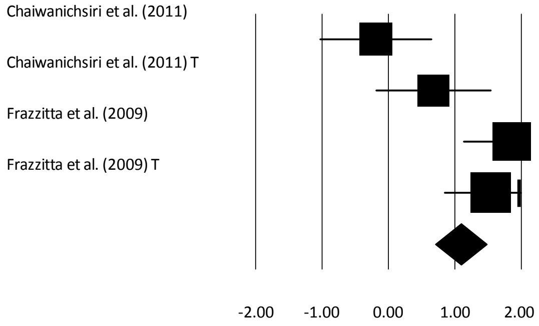

Figure 11 Gait velocity analysed with training for 20 minutes with rhythmic auditory cueing

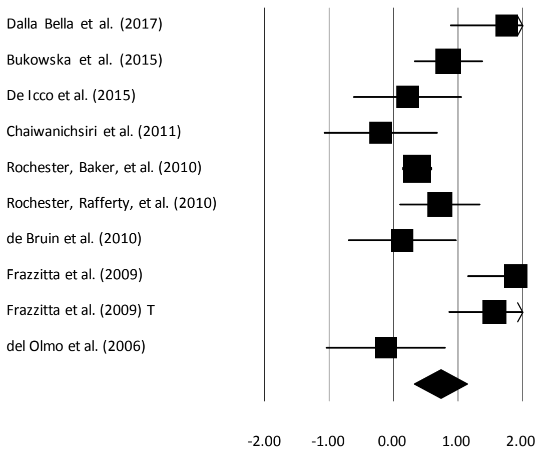

Figure 12 Gait velocity analysed with training for less than 5 weeks with rhythmic auditory cueing

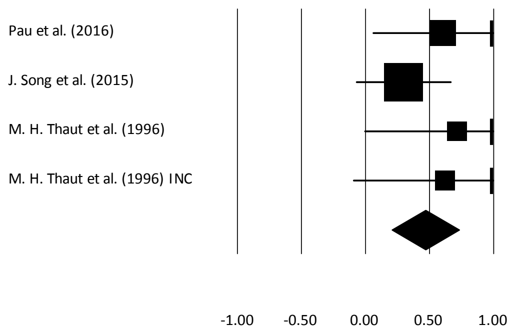

Figure 13 Gait velocity analysed with training for more than 5 weeks with rhythmic auditory cueing

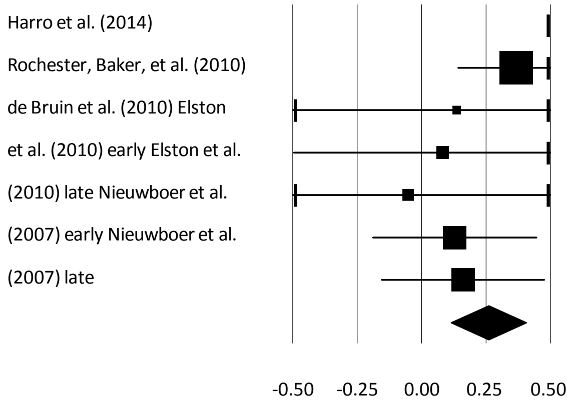

Figure 14 Gait velocity analysed in randomized controlled trials with rhythmic auditory cueing

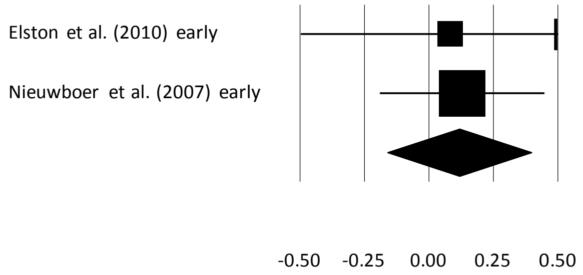

Figure 15 Gait velocity analysed in early group with rhythmic auditory cueing

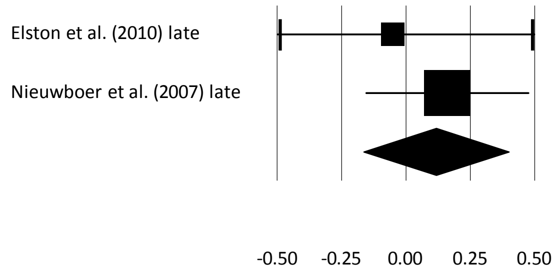

Figure 16 Gait velocity analysed in late group with rhythmic auditory cueing

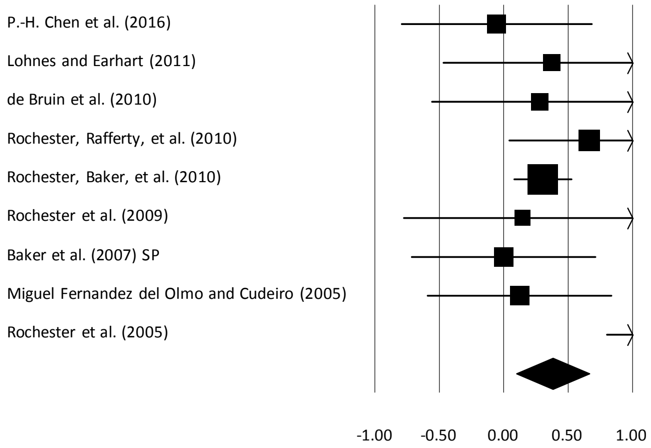

Figure 17 Gait velocity analysed with rhythmic auditory cueing and a dual task performed simultaneously

Stride length

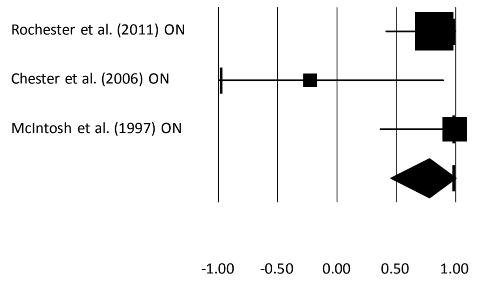

Figure 18 Stride length evaluated in ON medication condition with rhythmic auditory cueing

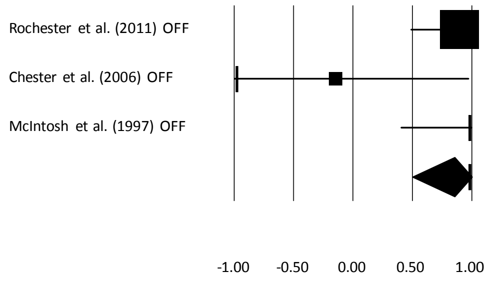

Figure 19 Stride length evaluated in ON medication condition with rhythmic auditory cueing

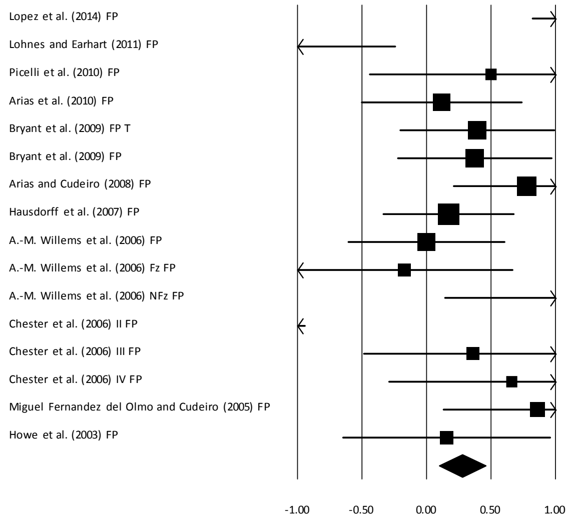

Figure 20 Stride length evaluated with fast paced rhythmic auditory cueing (pace of stimuli determined with reference to patient's preferred cadence)

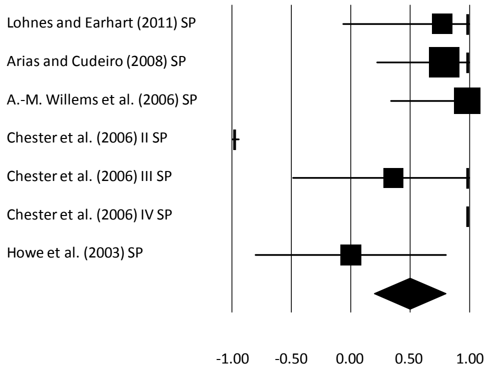

Figure 21 Stride length evaluated with slow paced rhythmic auditory cueing (pace of stimuli determined with reference to patient's preferred cadence)

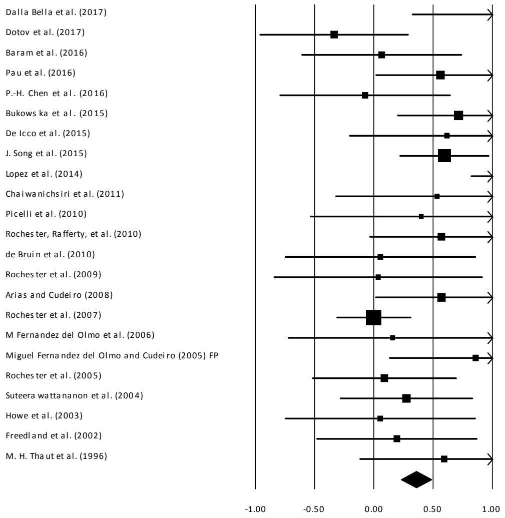

Figure 22 Stride length evaluated with un-modulated rhythmic auditory cueing

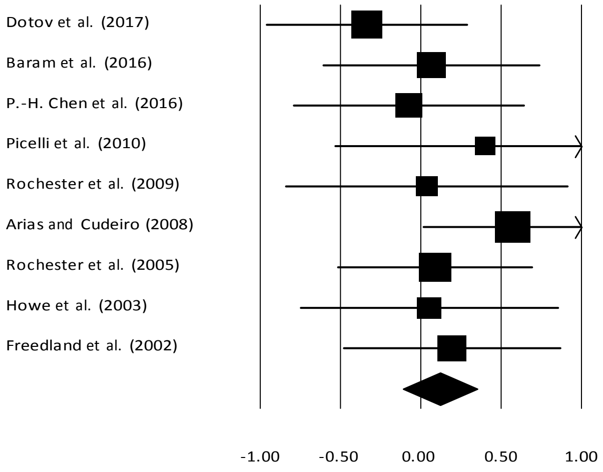

Figure 23 Stride length analysed with un-modulated rhythmic auditory cueing without training

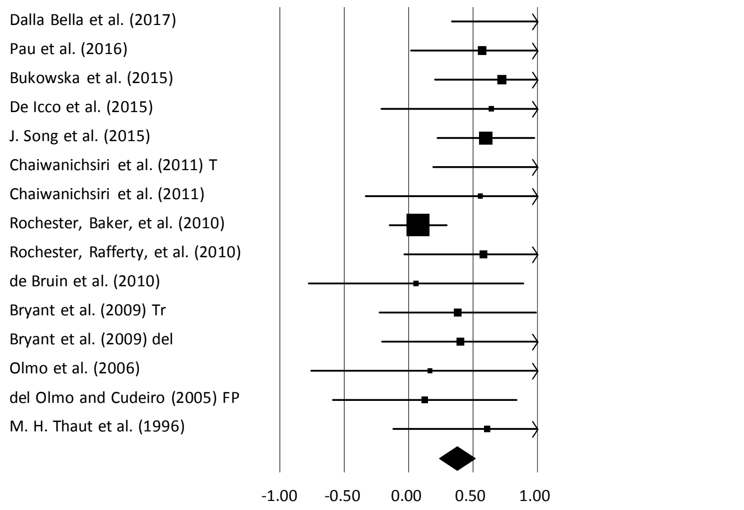

Figure 24 Stride length analysed with rhythmic auditory cueing with training

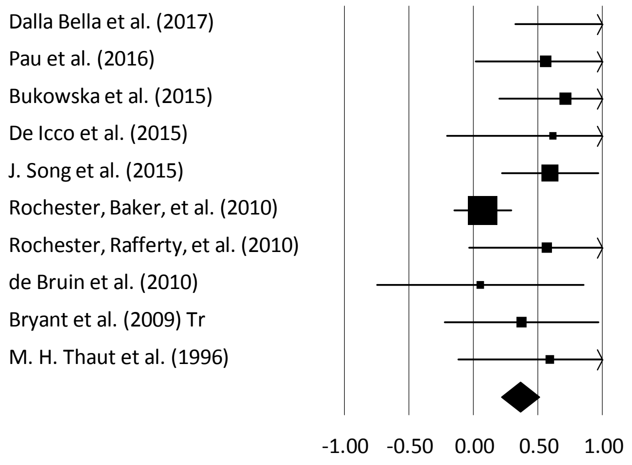

Figure 25 Stride length analysed with training for 30 minutes with rhythmic auditory cueing

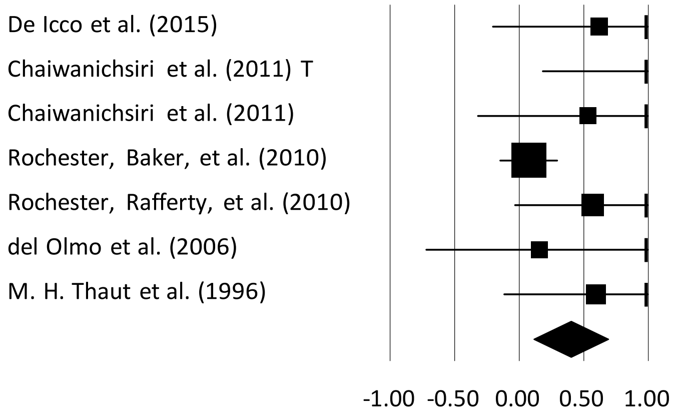

Figure 26 Stride length analysed with training for more than 5 sessions per week with rhythmic auditory cueing

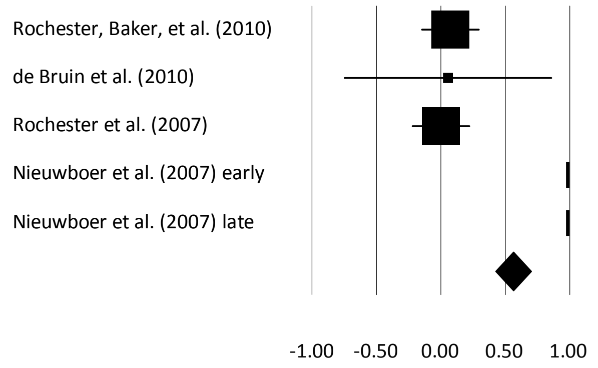

Figure 27 Stride length analysed in randomized controlled trials with rhythmic auditory cueing

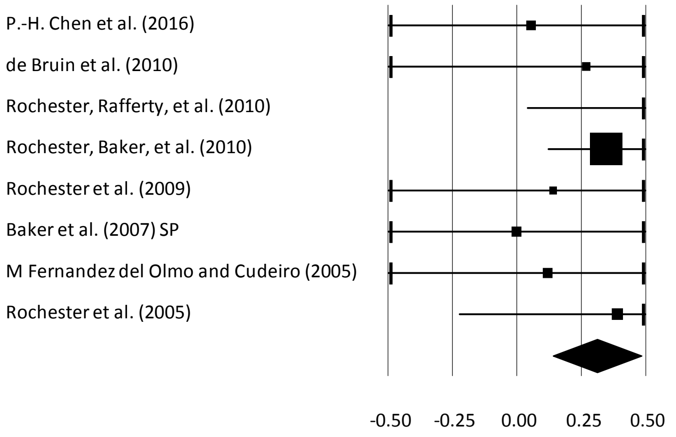

Figure 28 Stride length analysed with rhythmic auditory cueing and a dual task performed simultaneously

Cadence

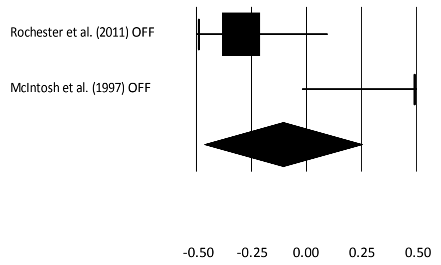

Figure 29 Cadence evaluated in OFF medication condition with rhythmic auditory cueing

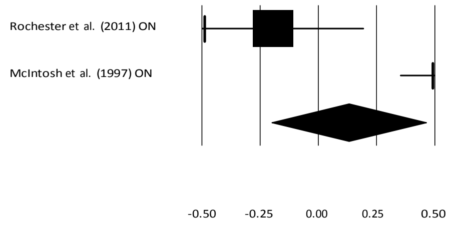

Figure 30 Cadence evaluated in ON medication condition with rhythmic auditory cueing

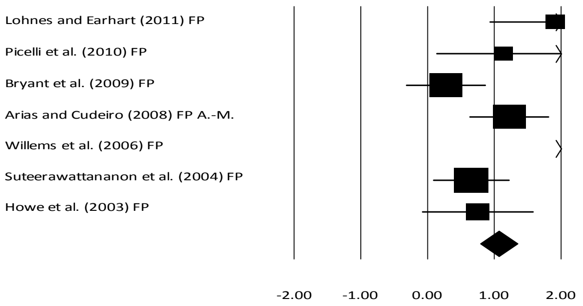

Figure 31 Cadence evaluated with fast paced rhythmic auditory cueing (pace of stimuli determined with reference to patient's preferred cadence)

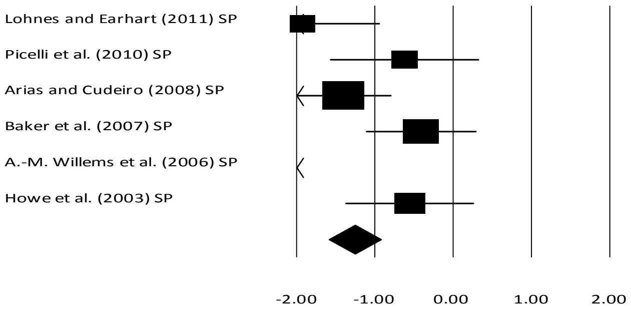

Figure 32 Cadence evaluated with slow paced rhythmic auditory cueing (pace of stimuli determined with reference to patient's preferred cadence)

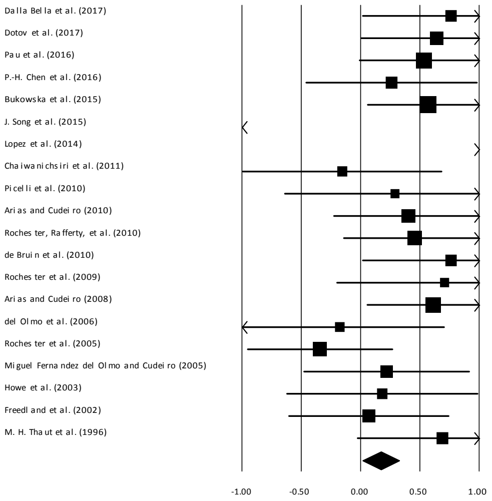

Figure 33 Cadence evaluated with un-modulated rhythmic auditory cueing

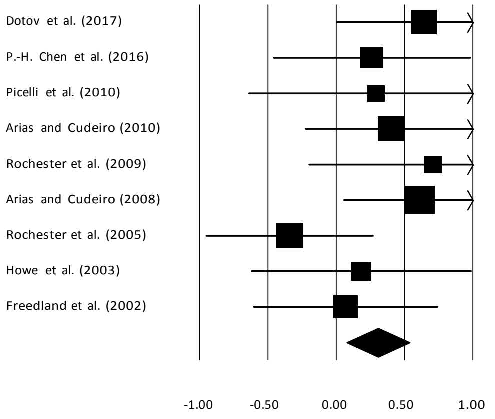

Figure 34 Cadence analysed with un-modulated rhythmic auditory cueing without training

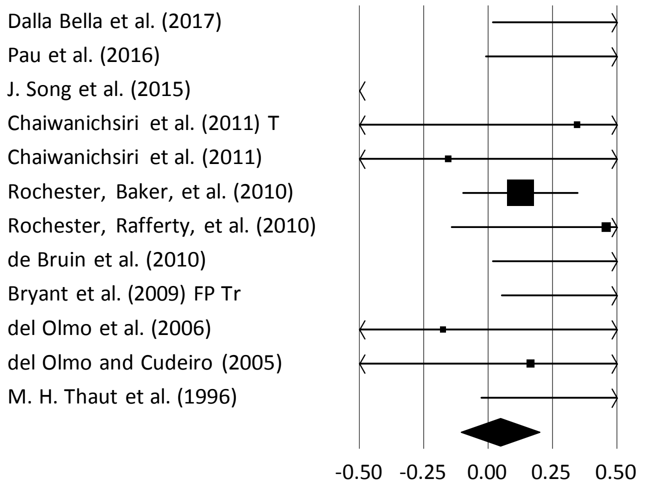

Figure 35 Cadence analysed with rhythmic auditory cueing with training

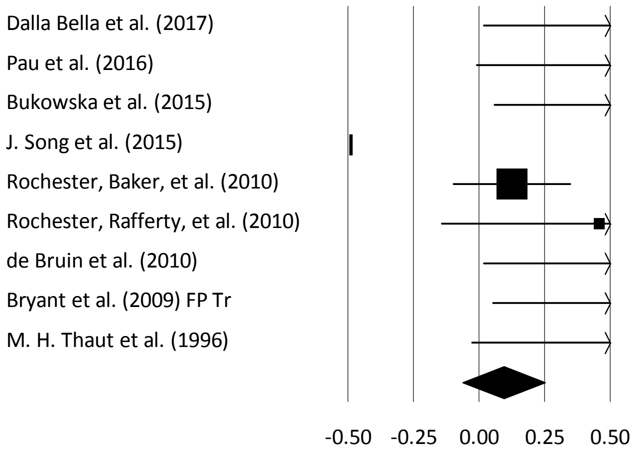

Figure 36 Cadence evaluated with training for 30 minutes with rhythmic auditory cueing

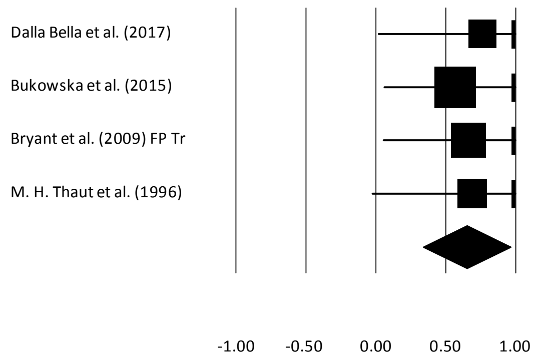

Figure 37 Cadence evaluated with training for less than 5 weeks training with rhythmic auditory cueing

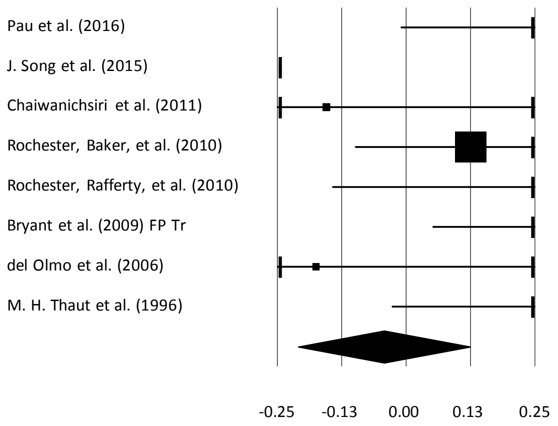

Figure 38 Cadence evaluated with training for more than 5 weeks training with rhythmic auditory cueing

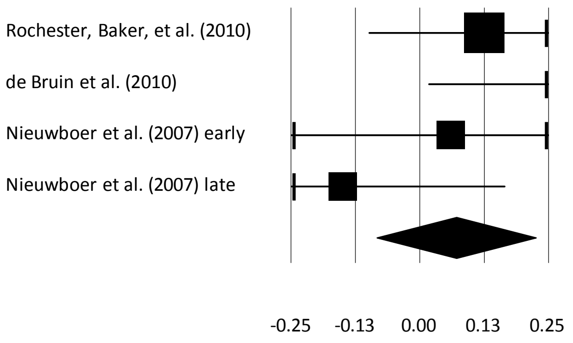

Figure 39 Cadence analysed in randomized controlled trials with rhythmic auditory cueing

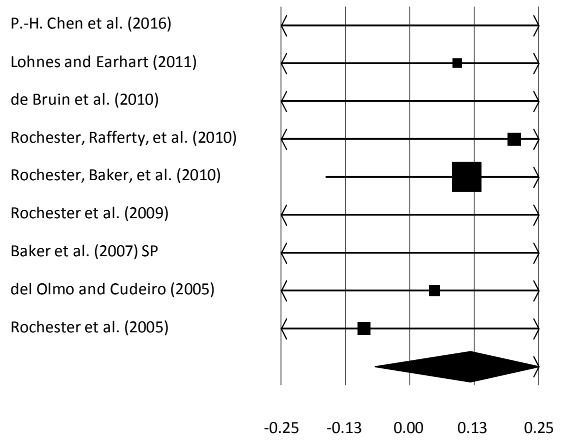

Figure 40 Cadence analysed with rhythmic auditory cueing and a dual task performed simultaneously

Double limb support

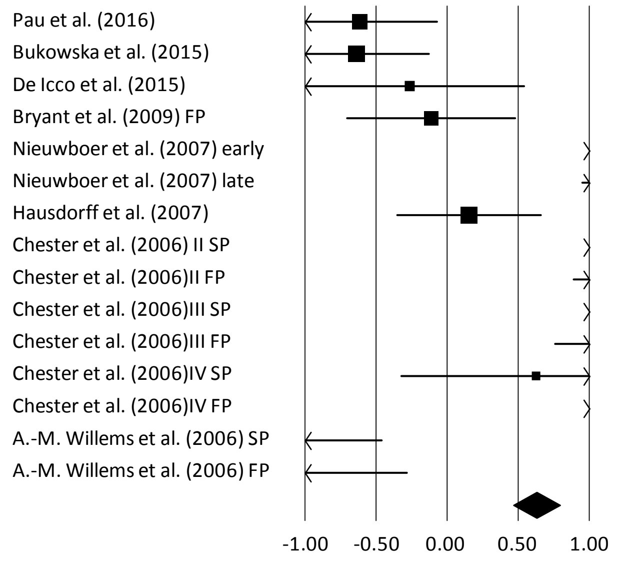

Figure 41 Double limb support duration analysed with rhythmic auditory cueing

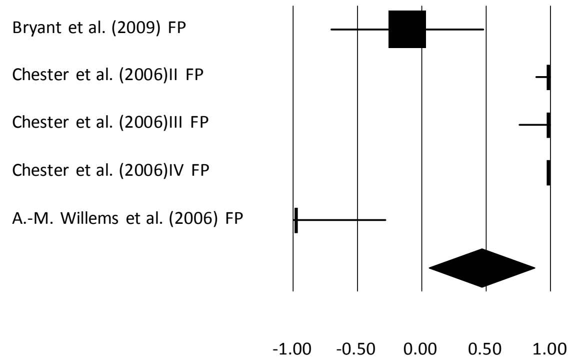

Figure 42 Double limb support duration analysed with fast paced rhythmic auditory cueing (reference patient's preferred cadence)

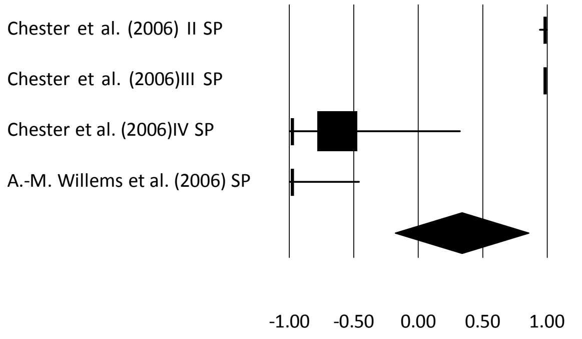

Figure 43 Double limb support duration analysed with slow paced rhythmic auditory cueing (reference patient's preferred cadence)

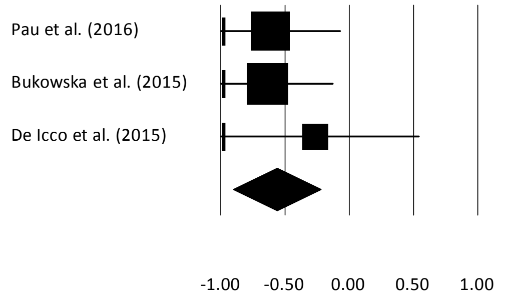

Figure 44 Double limb support duration analysed with un-modulated rhythmic auditory cueing

Turn time

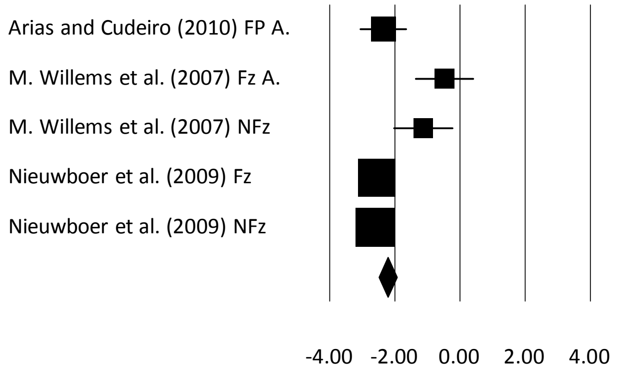

Figure 45 Turn time analysed with rhythmic auditory cueing

References

1. Dotov, D. *et al.* Biologically-variable rhythmic auditory cues are superior to isochronous cues in fostering natural gait variability in Parkinson's disease. *Gait Posture* **51**, 64-69 (2017).
2. Dalla Bella, S. *et al.* Gait improvement via rhythmic stimulation in Parkinson's disease is linked to rhythmic skills. *Scientific Reports* **7** (2017).
3. Chen, P.-H. *et al.* Walking Turns in Parkinson's Disease Patients with Freezing of Gait: The Short-term Effects of Different Cueing Strategies. *International Journal of Gerontology* **10**, 71-75 (2016).
4. Pau, M. *et al.* effects of Physical rehabilitation integrated with rhythmic auditory stimulation on spatio-Temporal and Kinematic Parameters of gait in Parkinson's Disease. *Frontiers in neurology* **7** (2016).
5. Zhao, Y. *et al.* Feasibility of external rhythmic cueing with the Google Glass for improving gait in people with Parkinson's disease. *Journal of neurology* **263**, 1156-1165 (2016).
6. Baram, Y., Aharon-Peretz, J., Badarny, S., Susel, Z. & Schlesinger, I. Closed-loop auditory feedback for the improvement of gait in patients with Parkinson's disease. *Journal of the neurological sciences* **363**, 104-106 (2016).
7. Son, H. & Kim, E. Kinematic analysis of arm and trunk movements in the gait of Parkinson's disease patients based on external signals. *Journal of physical therapy science* **27**, 3783-3786 (2015).
8. Song, J. *et al.* Rhythmic auditory stimulation with visual stimuli on motor and balance function of patients with Parkinson's disease. *Eur Rev Med Pharmacol Sci* **19**, 2001-2007 (2015).
9. De Icco, R. *et al.* Acute and chronic effect of acoustic and visual cues on gait training in Parkinson's disease: a randomized, controlled study. *Parkinson's Disease* **2015** (2015).
10. Bukowska, A. A., Krężałek, P., Mirek, E., Bujas, P. & Marchewka, A. Neurologic music therapy training for mobility and stability rehabilitation with Parkinson's disease–A pilot study. *Frontiers in human neuroscience* **9** (2015).
11. Benoit, C.-E. *et al.* Musically cued gait-training improves both perceptual and motor timing in Parkinson's disease. *Frontiers in human neuroscience* **8**, 494 (2014).
12. Harro, C. *et al.* The effects of speed-dependent treadmill training and rhythmic auditory-cued overground walking on balance function, fall incidence, and quality of life in individuals with idiopathic Parkinson's disease: A randomized controlled trial. *NeuroRehabilitation* **34**, 541-556 (2014).
13. Lopez, W. O. *et al.* Listenmee and Listenmee smartphone application: synchronizing walking to rhythmic auditory cues to improve gait in Parkinson's disease. *Hum Mov Sci* **37**, 147-156, doi:10.1016/j.humov.2014.08.001 (2014).
14. Young, W. R., Rodger, M. W. & Craig, C. M. Auditory observation of stepping actions can cue both spatial and temporal components of gait in Parkinson′ s disease patients. *Neuropsychologia* **57**, 140-153 (2014).
15. Hove, M. J., Suzuki, K., Uchitomi, H., Orimo, S. & Miyake, Y. Interactive rhythmic auditory stimulation reinstates natural 1/f timing in gait of Parkinson's patients. *PloS one* **7**, e32600 (2012).
16. Kadivar, Z., Corcos, D. M., Foto, J. & Hondzinski, J. M. Effect of step training and rhythmic auditory stimulation on functional performance in Parkinson patients. *Neurorehabilitation and neural repair* **25**, 626-635 (2011).
17. Rochester, L., Baker, K., Nieuwboer, A. & Burn, D. Targeting dopa-sensitive and dopa-resistant gait dysfunction in Parkinson's disease: Selective responses to internal and external cues. *Movement Disorders* **26**, 430-435 (2011).

18 Lohnes, C. A. & Earhart, G. M. The impact of attentional, auditory, and combined cues on walking during single and cognitive dual tasks in Parkinson disease. *Gait Posture* **33**, 478-483 (2011).
19 Ford, M. P., Malone, L. A., Nyikos, I., Yelisetty, R. & Bickel, C. S. Gait training with progressive external auditory cueing in persons with Parkinson's disease. *Archives of physical medicine and rehabilitation* **91**, 1255-1261 (2010).
20 Espay, A. J. *et al.* At-home training with closed-loop augmented-reality cueing device for improving gait in patients with Parkinson disease. *Journal of rehabilitation research and development* **47**, 573 (2010).
21 Lim, I. *et al.* Does cueing training improve physical activity in patients with Parkinson’s disease? *Neurorehabilitation and Neural Repair* **24**, 469-477 (2010).
22 Arias, P. & Cudeiro, J. Effect of rhythmic auditory stimulation on gait in Parkinsonian patients with and without freezing of gait. *PloS one* **5**, e9675 (2010).
23 Chaiwanichsiri, D., Wangno, W., Kitisomprayoonkul, W. & Bhidayasiri, R. Treadmill training with music cueing: a new approach for Parkinson’s gait facilitation. (2011).
24 de Bruin, N. *et al.* Walking with music is a safe and viable tool for gait training in Parkinson's disease: the effect of a 13-week feasibility study on single and dual task walking. *Parkinson’s disease* **2010** (2010).
25 Elston, J., Honan, W., Powell, R., Gormley, J. & Stein, K. Do metronomes improve the quality of life in people with Parkinson’s disease? A pragmatic, single-blind, randomized cross-over trial. *Clin Rehabil* **24**, 523-532 (2010).
26 Rochester, L. *et al.* Evidence for motor learning in Parkinson's disease: acquisition, automaticity and retention of cued gait performance after training with external rhythmical cues. *Brain research* **1319**, 103-111 (2010).
27 Picelli, A. *et al.* Three-dimensional motion analysis of the effects of auditory cueing on gait pattern in patients with Parkinson’s disease: a preliminary investigation. *Neurological Sciences* **31**, 423-430 (2010).
28 Rochester, L. *et al.* The effect of cueing therapy on single and dual-task gait in a drug naïve population of people with Parkinson's disease in northern Tanzania. *Movement Disorders* **25**, 906-911 (2010).
29 Frazzitta, G., Maestri, R., Uccellini, D., Bertotti, G. & Abelli, P. Rehabilitation treatment of gait in patients with Parkinson's disease with freezing: a comparison between two physical therapy protocols using visual and auditory cues with or without treadmill training. *Movement Disorders* **24**, 1139-1143 (2009).
30 Rochester, L., Burn, D. J., Woods, G., Godwin, J. & Nieuwboer, A. Does auditory rhythmical cueing improve gait in people with Parkinson's disease and cognitive impairment? A feasibility study. *Movement Disorders* **24**, 839-845 (2009).
31 Bryant, M., Rintala, D., Lai, E. & Protas, E. An evaluation of self-administration of auditory cueing to improve gait in people with Parkinson’s disease. *Clin Rehabil* **23**, 1078-1085 (2009).
32 Ma, H.-I., Hwang, W.-J. & Lin, K.-C. The effects of two different auditory stimuli on functional arm movement in persons with Parkinson's disease: a dual-task paradigm. *Clin Rehabil* **23**, 229-237 (2009).
33 Nieuwboer, A. *et al.* The short-term effects of different cueing modalities on turn speed in people with Parkinson's disease. *Neurorehabilitation and neural repair* (2009).
34 Arias, P. & Cudeiro, J. Effects of rhythmic sensory stimulation (auditory, visual) on gait in Parkinson’s disease patients. *Exp Brain Res* **186**, 589-601 (2008).
35 Baker, K., Rochester, L. & Nieuwboer, A. The effect of cues on gait variability—Reducing the attentional cost of walking in people with Parkinson's disease. *Parkinsonism & related disorders* **14**, 314-320 (2008).

36 Rochester, L. *et al.* The attentional cost of external rhythmical cues and their impact on gait in Parkinson's disease: effect of cue modality and task complexity. *Journal of neural transmission* **114**, 1243 (2007).
37 Baker, K., Rochester, L. & Nieuwboer, A. The immediate effect of attentional, auditory, and a combined cue strategy on gait during single and dual tasks in Parkinson's disease. *Archives of physical medicine and rehabilitation* **88**, 1593-1600 (2007).
38 Hausdorff, J. M. *et al.* Rhythmic auditory stimulation modulates gait variability in Parkinson's disease. *European Journal of Neuroscience* **26**, 2369-2375 (2007).
39 Nieuwboer, A. *et al.* Cueing training in the home improves gait-related mobility in Parkinson's disease: the RESCUE trial. *Journal of Neurology, Neurosurgery & Psychiatry* **78**, 134-140 (2007).
40 Willems, A. M. *et al.* Turning in Parkinson's disease patients and controls: the effect of auditory cues. *Movement disorders* **22**, 1871-1878 (2007).
41 Chester, E. L., Turnbull, G. I. & Kozey, J. The effect of auditory cues on gait at different stages of parkinson's disease and during "on"/"off" fluctuations: a preliminary study. *Topics in Geriatric Rehabilitation* **22**, 187-195 (2006).
42 Willems, A.-M. *et al.* The use of rhythmic auditory cues to influence gait in patients with Parkinson's disease, the differential effect for freezers and non-freezers, an explorative study. *Disability and rehabilitation* **28**, 721-728 (2006).
43 del Olmo, M. F., Arias, P., Furio, M., Pozo, M. & Cudeiro, J. Evaluation of the effect of training using auditory stimulation on rhythmic movement in Parkinsonian patients—a combined motor and [18 F]-FDG PET study. *Parkinsonism & related disorders* **12**, 155-164 (2006).
44 Jiang, Y. & Norman, K. E. Effects of visual and auditory cues on gait initiation in people with Parkinson's disease. *Clin Rehabil* **20**, 36-45, doi:10.1191/0269215506cr925oa (2006).
45 del Olmo, M. F. & Cudeiro, J. Temporal variability of gait in Parkinson disease: Effectsof a rehabilitation programme based on rhythmic sound cues. *Parkinsonism & related disorders* **11**, 25-33 (2005).
46 Rochester, L. *et al.* The effect of external rhythmic cues (auditory and visual) on walking during a functional task in homes of people with Parkinson's disease. *Archives of physical medicine and rehabilitation* **86**, 999-1006 (2005).
47 Suteerawattananon, M., Morris, G., Etnyre, B., Jankovic, J. & Protas, E. Effects of visual and auditory cues on gait in individuals with Parkinson's disease. *Journal of the neurological sciences* **219**, 63-69 (2004).
48 Cubo, E., Leurgans, S. & Goetz, C. G. Short-term and practice effects of metronome pacing in Parkinson's disease patients with gait freezing while in the 'on'state: randomized single blind evaluation. *Parkinsonism & related disorders* **10**, 507-510 (2004).
49 Howe, T. E., Lövgreen, B., Cody, F. W., Ashton, V. & Oldham, J. Auditory cues can modify the gait of persons with early-stage Parkinson's disease: a method for enhancing parkinsonian walking performance? *Clin Rehabil* **17**, 363-367 (2003).
50 Freedland, R. L. *et al.* The effects of pulsed auditory stimulation on various gait measurements in persons with Parkinson's disease. *NeuroRehabilitation* **17**, 81-87 (2002).
51 McIntosh, G. C., Brown, S. H., Rice, R. R. & Thaut, M. H. Rhythmic auditory-motor facilitation of gait patterns in patients with Parkinson's disease. *Journal of Neurology, Neurosurgery & Psychiatry* **62**, 22-26 (1997).
52 Thaut, M. H. *et al.* Rhythmic auditory stimulation in gait training for Parkinson's disease patients. *Movement disorders : official journal of the Movement Disorder Society* **11**, 193-200, doi:10.1002/mds.870110213 (1996).
